# Self-surface charge exfoliation and electrostatically coordinated 2D hetero-layered hybrids

**DOI:** 10.1038/ncomms14224

**Published:** 2017-02-01

**Authors:** Min-Quan Yang, Yi-Jun Xu, Wanheng Lu, Kaiyang Zeng, Hai Zhu, Qing-Hua Xu, Ghim Wei Ho

**Affiliations:** 1 Department of Electrical and Computer Engineering, National University of Singapore, 4 Engineering Drive 3, 117583 Singapore, Singapore; 2 State Key Laboratory of Photocatalysis on Energy and Environment, College of Chemistry, Fuzhou University, Fuzhou 350002, China; 3 College of Chemistry, New Campus, Fuzhou University, Fuzhou 350108, China; 4 Department of Mechanical Engineering, National University of Singapore, 9 Engineering Drive 1, 117576 Singapore, Singapore; 5 Department of Chemistry, National University of Singapore, 3 Science Drive 3, 117543 Singapore, Singapore

## Abstract

At present, the technological groundwork of atomically thin two-dimensional (2D) hetero-layered structures realized by successive thin film epitaxial growth is in principle constrained by lattice matching prerequisite as well as low yield and expensive production. Here, we artificially coordinate ultrathin 2D hetero-layered metal chalcogenides via a highly scalable self-surface charge exfoliation and electrostatic coupling approach. Specifically, bulk metal chalcogenides are spontaneously exfoliated into ultrathin layers in a surfactant/intercalator-free medium, followed by unconstrained electrostatic coupling with a dissimilar transition metal dichalcogenide, MoSe_2_, into scalable hetero-layered hybrids. Accordingly, surface and interfacial-dominated photocatalysis reactivity is used as an ideal testbed to verify the reliability of diverse 2D ultrathin hetero-layered materials that reveal high visible-light photoreactivity, efficient charge transfer and intimate contact interface for stable cycling and storage purposes. Such a synthetic approach renders independent thickness and composition control anticipated to advance the development of ‘design-and-build' 2D layered heterojunctions for large-scale exploration and applications.

## Abstract (web summary)

Synthesis of atomically thin 2D hetero-layered structures remains a challenge. Here, the authors report a scalable approach to fabricating 2D hetero-layered metal chalcogenides of various compositions: self-surface charge exfoliation, followed by electrostatic coupling.

Ultimate two-dimensional (2D) anisotropy with atomically thick layered structure is both an ideal low-dimensional system for fundamental study and an elemental building block for designed assembly^1^,^2^,^3^,^4^,^5^,^6^,^7^,^8^,^9^,^10^. Elaborate functionalities can rationally be tailored with precise molecular scale control through artificial assembly based on judicious selection and coordination of heterogeneous counterparts^11^,^12^,^13^,^14^. Intriguing surface effects and physical–chemical properties have gradually been uncovered in 2D hetero-layered materials owing to their large surface-to-volume ratio and confined thickness at an atomic scale^15^,^16^,^17^,^18^. The 2D materials, specifically metal chalcogenides, possess exquisite photo- and electrochemical capabilities that include energy storage batteries/supercapacitors and energy conversion photo/electrocatalysis systems^10^,^19^,^20^. Unlike electronic transistor devices, these applications generally require large quantities of 2D hetero-layered materials. Though a wide variety of 2D layered semiconductor materials have been exfoliated into individual layers, these layered materials typically undergo large extent of swelling phase induced by intercalator or solvation species^21^,^22^,^23^. Realization of high-volume exfoliation with ultrathin sheet-like crystallite in a facile manner and clean medium still remains scarce. Moreover, the issue of lattice mismatching hinders composition customization of heterostructures owing to the ineffective direct epitaxial growth of some mismatched metal chalcogenide materials. Collectively, all the aforementioned issues limit the scalability of 2D ultrathin hetero-layered metal chalcogenides towards fundamental exploration and advanced functional applications.

Herein, we readily exfoliate the metal chalcogenide semiconductor ZnIn_2_S_4_ into single-unit-cell layered structure (ca. 2.5 nm) via a self-surface charge exfoliation in pure water medium. Successive electrostatic coupling with another transition metal chalcogenide (for example, MoSe_2_) enables construction of arbitrary ultrathin hetero-layered hybrids in a large scale. Such an approach offers salient features, that is, independent thickness and composition control of individual layer assembly, and no constraint by lattice matching prerequisite into functional ultrathin heterostructures. Distinct emission lifetime reduction and photoluminescence quenching of the hetero-layered hybrid ascertain strong interlayer coupling and efficient charge transfer between the components. Surface and interfacial-dominated photocatalysis, a promising strategy for solar energy conversion^24^,^25^,^26^,^27^,^28^,^29^,^30^,^31^,^32^, is adopted to demonstrate the reliability of the catalytically rich 2D ultrathin ZnIn_2_S_4_/MoSe_2_ hetero-layered material. The as-synthesized ZnIn_2_S_4_/MoSe_2_ concurrently realize efficient separation and transfer of photogenerated charge carriers, acceleration of surface proton reduction with abundant active sites as well as enhanced visible light absorption that circumvent the limitations of conventional photocatalysts^33^. Consequently, the ZnIn_2_S_4_/MoSe_2_ displays high-performance visible-light-driven H_2_ evolution activity of 6,454 μmol g^−1^ h^−1^, ∼15 and 4 times as high as that of bulk and bare ZnIn_2_S_4_ nanosheets, respectively. Importantly, the 2D hetero-layered hybrid shows high stability with prolonged 80 h cycling and catalytic reactivity retention after storing over a few months that further attests to the integrity of the constructed 2D materials for prospective advanced applications. Furthermore, we test the applicability of this approach on other 2D metal sulfides, namely CdIn_2_S_4_ and In_2_S_3_ that also show scalable self-surface charge exfoliation. Similarly, the constructed hetero-layered hybrids of CdIn_2_S_4_/MoSe_2_ and In_2_S_3_/MoSe_2_ feature enhanced photocatalytic activity and stability.

## Results

### Self-surface charge exfoliation of ultrathin ZnIn_2_S_4_ layers

**Figure 1:**
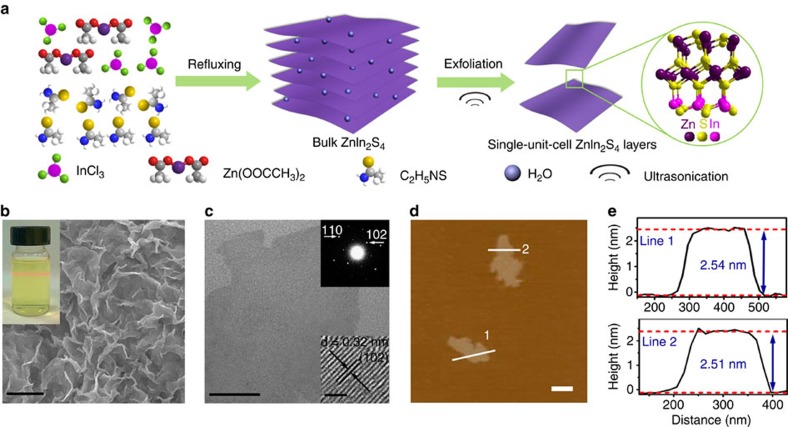
Schematic illustration of the synthesis of single-unit-cell ZnIn_2_S_4_ layers and characterization. (**a**) A self-surface charge promoted exfoliation of clean and freestanding single-unit-cell ZnIn_2_S_4_ layers in surfactant/intercalator-free medium. (**b**) Scanning electron microscopy (SEM) image of bulk ZnIn_2_S_4_. Scale bar, 2 μm (**c**) Transmission electron microscopy (TEM) image of single-unit-cell ZnIn_2_S_4_ layers. Scale bar, 50 nm. (**d**) Atomic force microscopy (AFM) image and (**e**) corresponding height images of single-unit-cell ZnIn_2_S_4_ layers. Scale bar, 200 nm. The inset in **b** is photograph of Tyndall effect of the ZnIn_2_S_4_ suspension; insets in **c** are the corresponding selected-area electron diffraction (SAED) pattern and high-resolution TEM (HRTEM) image of single-unit-cell ZnIn_2_S_4_ layers.

The ultrathin single-unit-cell ZnIn_2_S_4_ layers were prepared via a facile low-temperature refluxing method, followed by a water-assisted surfactant/intercalator-free exfoliation process, as schematically illustrated in Fig. 1a (for more details, see Methods). Scanning electron microscopy (SEM) images of bulk ZnIn_2_S_4_ in Fig. 1b and Supplementary Fig. 1 reveal a uniform morphology of sheet-like structure. The energy-dispersive X-ray spectroscopy (EDX) (Supplementary Fig. 2) and X-ray diffraction (XRD) (Supplementary Fig. 3) analysis confirm the elemental composition and high purity of the as-synthesized ZnIn_2_S_4_ with hexagonal phase structure (cell parameters of *a*=*b*=3.85 Å, *c*=24.68 Å, JCPDS No. 65–2,023).

Notably, the dispersion of ZnIn_2_S_4_ in deionized (DI) water reveals a strong negatively charged surface with a zeta potential value of −36.5 mV (Supplementary Fig. 4). This can be ascribed to the presence of excess amount of S^2−^ that has been adsorbed onto the ZnIn_2_S_4_ surface during the synthesis process^34^,^35^,^36^. Other supporting evidences are appended in Supplementary Figs 5 and 6. The self-surface charge within the layered nanostructure significantly weakens the interaction between ZnIn_2_S_4_ interlayers. Owing to Coulombic repulsion, these layers repel each other and are readily exfoliated with the assistance of shear forces triggered by mild sonication in the absence of any intercalator and surfactant species (Supplementary Fig. 7).

Transmission electron microscopy (TEM) images (Fig. 1c and Supplementary Fig. 8) of the as-exfoliated ZnIn_2_S_4_ layers display 2D sheet structure with a nearly transparent feature, implying the ultrathin nature of the exfoliated product. This self-surface charge promoted exfoliation can be corroborated by the controlled experiment of ultrasonic exfoliation of ZnIn_2_S_4_ synthesized from the addition of a stoichiometric amount of S^2−^ (denoted as ZnIn_2_S_4_-S, see Methods for more details), as illustrated in Supplementary Fig. 9. The as-obtained ZnIn_2_S_4_-S displays a weak zeta potential of −5.2 mV (Supplementary Fig. 10). Accordingly, the dispersion of ZnIn_2_S_4_-S can be easily centrifuged after ultrasonication. The TEM image in Supplementary Fig. 11 demonstrates that the ZnIn_2_S_4_-S is composed of aggregated layers. In addition, negatively charged ZnIn_2_S_4_ surface can also be validated by a layer electrostatic self-assembly demonstration of the ZnIn_2_S_4_ nanosheets on positively charged 3-aminopropyl-triethoxysilane (APTES)-modified glass substrate (Supplementary Figs 12 and 13). Correspondingly, a pale yellow thin film can be observed only for the strong negatively charged ZnIn_2_S_4_ (−36.5 mV) coating on the glass surface. SEM image reveals uniform coverage/assembly of strong negatively charged ZnIn_2_S_4_ nanosheets on the positive APTES-glass substrate. Conversely, no obvious adsorption of the weak negatively charged ZnIn_2_S_4_-S (−5.2 mV) has been observed on the glass substrate. Altogether, these findings confirm the existence of strong negatively self-charged ZnIn_2_S_4_ surface that facilitates facile interlayer exfoliation of ultrathin ZnIn_2_S_4_ nanosheets without the assistance of surfactant/intercalator additives.

Moreover, the corresponding selected-area electron diffraction pattern in Fig. 1c inset shows clear bright spots that correspond to the hexagonal structure and single-crystalline characteristic of the ultrathin ZnIn_2_S_4_ nanosheets. In addition, the high-resolution TEM (HRTEM) image in Fig. 1c inset displays distinct lattice fringes of ca. 0.32 nm, corresponding to the (102) crystallographic plane of ZnIn_2_S_4_. The typical Tyndall effect observed for the as-exfoliated ZnIn_2_S_4_ suspension using a red laser (Fig. 1b, inset) indicates the formation of freestanding and highly dispersed ultrathin ZnIn_2_S_4_ layers. More importantly, the colloid ultrathin ZnIn_2_S_4_ nanosheets can be easily exfoliated into large scale (Supplementary Fig. 14) that is essential for further utilization.

TEM characterization cannot unambiguously determine the ultimate thickness of the layers. In this context, atomic force microscopy (AFM) is used to provide an estimated quantitative layer thickness. As shown in Fig. 1d, the topography of the as-prepared ZnIn_2_S_4_ presents 2D structure with smooth surface. The corresponding height profiles in Fig. 1e show that the typical thickness of the as-exfoliated layers is ∼2.5 nm, validating the ultrathin nature of the ZnIn_2_S_4_. Considering that the *c* parameter of ZnIn_2_S_4_ is 24.68 Å (Supplementary Fig. 15), the thickness of ZnIn_2_S_4_ is well in agreement with the thickness of a unit cell along the [001] axis^37^. Thus, it is reasonable to deduce that each ultrathin ZnIn_2_S_4_ nanosheet with a thickness of ca. 2.5 nm is a single-unit-cell ZnIn_2_S_4_ atomic layer. According to the diffusion formula of *t*=*d*^2^/*k*^2^*D* (*d* is the particle size, *k* is a constant, *D* is the diffusion coefficient of electron–hole pairs)^5^,^7^, the ultrathin single-unit-cell ZnIn_2_S_4_ atomic layer will significantly shorten the diffusion length and time of charge carriers taken to reach the surface, enable the photoexcited electron–hole pairs to transport from the interior to the surface fast, and thus lead to higher charge separation efficiency of the ultrathin ZnIn_2_S_4_ than its counterpart of bulk ZnIn_2_S_4_, as verified by the photocurrent and photoluminescence (PL) analysis in Supplementary Figs 16 and 17.

### Generalized synthesis of ultrathin CdIn_2_S_4_ and In_2_S_3_ layers

The suitability of self-surface charge exfoliation as a general approach for large-scale preparation of other 2D metal sulfides, that is, CdIn_2_S_4_ and In_2_S_3_, has also been demonstrated. As shown in Supplementary Fig. 18, the CdIn_2_S_4_ dispersion in DI water shows a strong negative charge with a zeta potential value of −39.7 mV. TEM images in Supplementary Fig. 19 demonstrate the 2D sheet structure of the exfoliated CdIn_2_S_4_ nanosheets without using any surfactant or intercalator under moderate ultrasonication. The inset in Supplementary Fig. 19 shows HRTEM image where the distinct lattice fringes of 0.33 nm correspond to the (311) crystallographic plane of CdIn_2_S_4_. AFM topography reveals the ultrathin structure of the exfoliated CdIn_2_S_4_ with the thickness of ∼2.3 nm (Supplementary Fig. 20). Typical Tyndall effect is observed for the as-exfoliated CdIn_2_S_4_ suspension (Supplementary Fig. 21), indicating the formation of large-scale freestanding and highly dispersed CdIn_2_S_4_ layers. Moreover, the respective EDX and XRD analyses in Supplementary Figs 22 and 23 confirm the as-synthesized CdIn_2_S_4_ nanosheets with cubic phase structure (JCPDS No. 27-0060).

Likewise, this self-surface charge strategy is also exploited to exfoliate In_2_S_3_ into a large-scale colloidal dispersion of ultrathin nanosheets (Supplementary Fig. 24). Zeta potential measurement displays a strong negatively charged surface (zeta potential of −41.9 mV) of In_2_S_3_ (Supplementary Fig. 25). TEM images in Supplementary Fig. 26 show 2D sheet structure with distinct lattice fringes (ca. 0.32 nm) of In_2_S_3_ (311) crystallographic plane. The thickness of the obtained In_2_S_3_ is ∼4.5 nm (Supplementary Fig. 27). Moreover, EDX result confirms the elemental composition of In_2_S_3_ and XRD pattern reveals the pure cubic phase of the synthesized In_2_S_3_ (JCPDS No. 65-0459) (Supplementary Figs 28 and 29).

### Construction of hetero-layered hybrids

Recent advances in creating heterostructures based on 2D atomic crystals by artificial combination of various 2D materials have shown to strongly modulate electronic and optical properties^17^. By virtue of the 2D configuration with large surface area, along with its complementary high interfacial contact with other components, the single-unit-cell ZnIn_2_S_4_ layers provide a favourable platform for the fabrication of hybrid composite^17^,^38^. In this context, MoSe_2_ nanosheets are integrated with the ultrathin ZnIn_2_S_4_ via a surface charge promoted self-assembly method that is driven by strong electrostatic attraction between the negatively charged ZnIn_2_S_4_ layers and positively charged MoSe_2_ (see Methods for more details). The electrostatic self-assembly method efficiently circumvents the requirement of lattice matching of two individual layers for assembling hetero-layer structure^39^,^40^. Consequently, a large quantity of hetero-layered ZnIn_2_S_4_/MoSe_2_ can be facilely obtained (Supplementary Fig. 30). Moreover, this method also imparts strong hetero-interlayer coupling that effectively promotes interfacial charge carriers transfer^41^,^42^,^43^ that will be discussed later.

**Figure 2:**
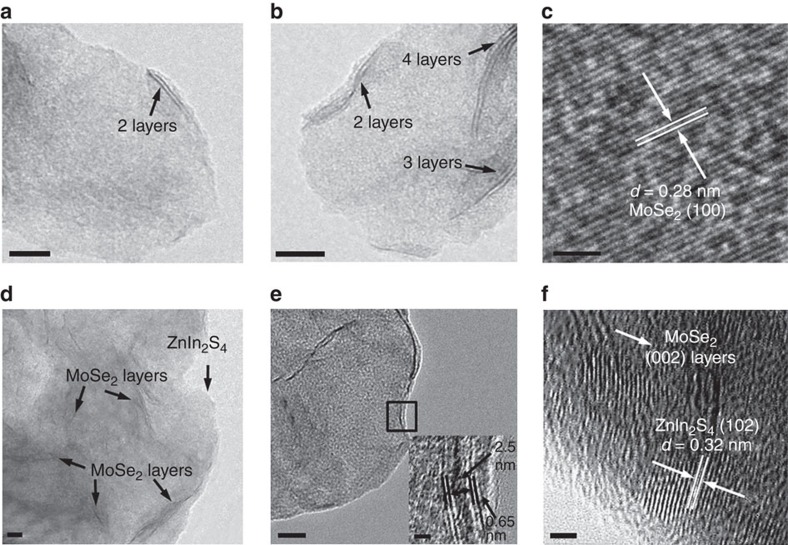
Morphology and structure of few-layered MoSe_2_ and hetero-layered ZnIn_2_S_4_/MoSe_2_. (**a**,**b**,**d**,**e**) Transmission electron microscopy (TEM) images of few-layered MoSe_2_ (**a**,**b**) and hetero-layered ZnIn_2_S_4_/MoSe_2_ (**d**,**e**). Scale bar, 10 nm. (**c**,**f**) High-resolution TEM (HRTEM) images of MoSe_2_ (**c**) and ZnIn_2_S_4_/MoSe_2_ (**f**). Scale bar, 2 nm. The inset in **e** is the magnification of the image shown in the black box of Fig. 2e. Scale bar, 2 nm.

**Figure 3:**
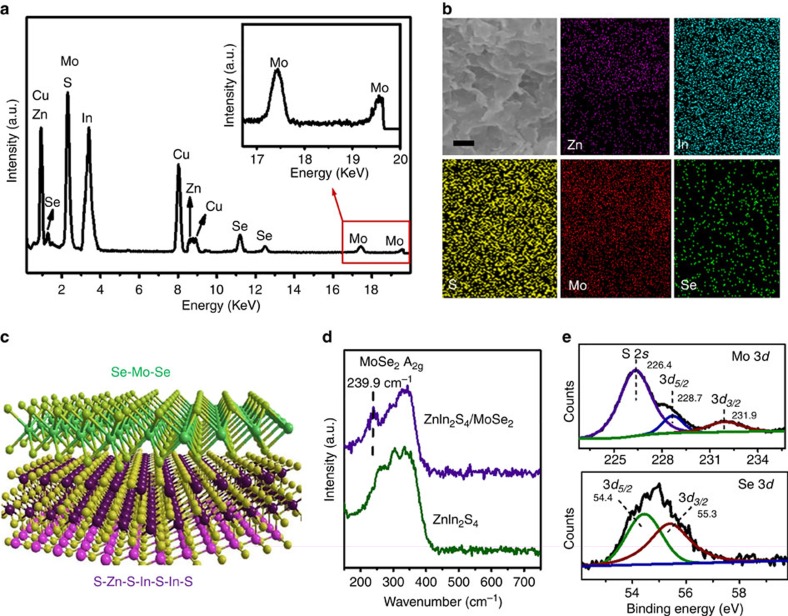
Characterization of hetero-layered ZnIn_2_S_4_/MoSe_2_. (**a**) Energy-dispersive X-ray (EDX) spectrum and (**b**) mapping images of the as-prepared ZnIn_2_S_4_/MoSe_2_. Scale bar, 200 nm. (**c**) Schematic illustration of the sheet-on-sheet ZnIn_2_S_4_/MoSe_2_ hetero-layer structure. (**d**) Raman spectra of ZnIn_2_S_4_ and ZnIn_2_S_4_/MoSe_2_. (**e**) High-resolution X-ray photoelectron spectroscopy (XPS) spectra of Mo 3*d* and Se 3*d*.

Figure 2a,b shows the TEM images of MoSe_2_, indicating that the sheets are typically composed of 2–4 layers with an interlayer spacing of 0.65 nm that corresponds to the (002) plane of hexagonal MoSe_2_ (refs 44, 45). The HRTEM image in Fig. 2c shows a *d* spacing of 0.28 nm that matches with the interspacing of (100) MoSe_2_ (refs 46, 47). After the integration of MoSe_2_ with the single-unit-cell ZnIn_2_S_4_ layers, the as-prepared ZnIn_2_S_4_/MoSe_2_ yields a hetero-layered structure (Fig. 2d,e). Figure 2f and Supplementary Fig. 31 show HRTEM images of ZnIn_2_S_4_/MoSe_2_ that display interlayer spacing of MoSe_2_ (0.65 nm) and distinct lattice fringes of ZnIn_2_S_4_ (0.32 nm), confirming the co-existence and interfacial contact of the two components. In addition, Supplementary Fig. 32 shows overlapping TEM mapping of various elements, also indicating the lamellar structure formation of vertically coordinated ZnIn_2_S_4_ and MoSe_2_ heterostructure. The corresponding EDX spectrum (Fig. 3a) shows the coexistence of Zn, In, S, Mo and Se elements, whereas the EDX mapping (Fig. 3b) demonstrates the homogeneous distribution of these elements throughout the ZnIn_2_S_4_/MoSe_2_ composite. Therefore, based on the above analyses, it is reasonable to infer that the ultrathin single-unit-cell ZnIn_2_S_4_ layers are intimately coupled with the layered MoSe_2_ featuring a sheet-on-sheet hetero-layer structure, as schematically reflected in Fig. 3c.

Moreover, the XRD patterns of ZnIn_2_S_4_ and ZnIn_2_S_4_/MoSe_2_ composite (Supplementary Fig. 33) present analogous diffraction peaks of hexagonal phase ZnIn_2_S_4_. The absence of typical MoSe_2_ peaks (Supplementary Fig. 34) could be ascribed to the relatively low diffraction intensity of MoSe_2_ peaks shielded by the strong and broad peaks of ZnIn_2_S_4_. Notably, Fig. 3d shows the Raman spectra of ZnIn_2_S_4_ and ZnIn_2_S_4_/MoSe_2_ where a characteristic peak at 240 cm^−1^ corresponding to the ‘A_1g_' band of MoSe_2_ (refs 44, 48, 49) (Supplementary Fig. 35) is observed for ZnIn_2_S_4_/MoSe_2_ sample, confirming the formation of a composite with MoSe_2_ in the matrix of ZnIn_2_S_4_. In addition, the shift of the ‘A_1g_' band of ZnIn_2_S_4_/MoSe_2_ as compared with that of bare MoSe_2_ (241.1 cm^−1^) also implies the reduced layer aggregation of MoSe_2_ nanosheets in the hybrid composite^48^,^49^. The ultraviolet–visible (UV–vis) absorption spectra in Supplementary Fig. 36 show that the ZnIn_2_S_4_/MoSe_2_ displays enhancement in visible-light absorption as compared with ZnIn_2_S_4_. This can be attributed to the intrinsic background absorption of black-coloured MoSe_2_ (Supplementary Fig. 37). To further determine the composition and chemical states of the composite, the ZnIn_2_S_4_/MoSe_2_ has been characterized by X-ray photoelectron spectroscopy (XPS). The doublet peaks for Mo 3*d* at 228.7 and 231.9 eV (top panel of Fig. 3e) can be assigned to the Mo^+4^ valence state, whereas the peak at 226.4 eV should be assigned to S 2*s* (ref. 15). In the Se 3*d* XPS spectrum (bottom panel of Fig. 3e), the peaks at 54.4 and 55.3 eV are ascribed to Se^2−^ (ref. 47). Meanwhile, the high-resolution XPS spectra of Zn 2*p* peaks at 1021.8 and 1044.8 eV, In 3*d* peaks at 445.0 and 452.6 eV and S 2*p* at 161.9 and 163.0 eV (Supplementary Fig. 38) can be assigned to Zn^2+^, In^3+^ and S^2−^ of ZnIn_2_S_4_, respectively^50^,^51^,^52^. The XPS analysis corroborates the presence of MoSe_2_ in the ZnIn_2_S_4_/MoSe_2_ composite.

### Photoelectrochemical properties

**Figure 4:**
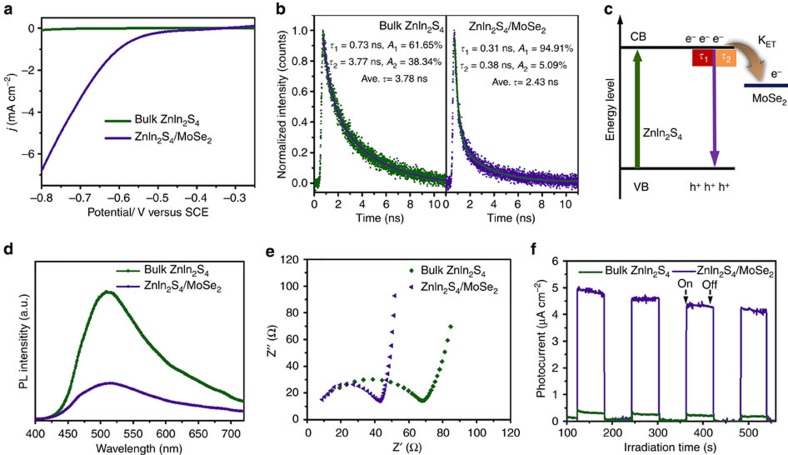
Photoelectrochemical properties. (**a**) Linear sweep voltammetry (LSV) curves, (**b**) time-resolved transient photoluminescence (PL) decay (excitation at 400 nm and emission at 495 nm), (**c**) schematic illustration of the interfacial charge carrier transfer, (**d**) steady-state PL spectra, (**e**) electrochemical impedance spectroscopy Nyquist plots and (**f**) transient photocurrent responses of bulk ZnIn_2_S_4_ and hetero-layered ZnIn_2_S_4_/MoSe_2_ composite. CB, conduction band; VB, valence band.

It has been well accepted that layered transition metal chalcogenides with exposed edge sites can effectively decrease activation energy/overpotential of redox reaction^17^ that are desirable for photo-/electrocatalytic processes. According to the energy band structures of ZnIn_2_S_4_ and MoSe_2_, the photogenerated electrons from the excitation of ZnIn_2_S_4_ are thermodynamically available for transferring to MoSe_2_. Therefore, it is anticipated that the ZnIn_2_S_4_/MoSe_2_ hetero-layered structure not only promotes charge separation and transport driven by junction/interface between the MoSe_2_ and the light harvester ZnIn_2_S_4_, but also facilitates the proton reduction on the surface of MoSe_2_. To gain more insight into its optoelectronic properties, a series of complementary photo- and electrochemical characterizations were carried out. Figure 4a displays the linear sweep voltammetry curves of ZnIn_2_S_4_/MoSe_2_ composite and bulk ZnIn_2_S_4_, revealing a higher cathodic current density that is attributed to the reduction of water to H_2_ (ref. 53) over hetero-layered ZnIn_2_S_4_/MoSe_2_ than bulk ZnIn_2_S_4_. Simultaneously, the controlled experiment over bare fluoride tin oxide (FTO) shows no obvious cathodic current under the same potential range (Supplementary Fig. 39). The result indicates that the integration of MoSe_2_ with ZnIn_2_S_4_ accelerates the protonation and subsequent H_2_ formation rate of ZnIn_2_S_4_/MoSe_2_ as compared with that of bulk ZnIn_2_S_4_.

In addition, Fig. 4b displays the time-resolved photoluminescence spectra of bulk ZnIn_2_S_4_ and hetero-layer structured ZnIn_2_S_4_/MoSe_2_, which probe the specific charge carrier dynamics of nanosystems^54^,^55^. The emission decay curves of the samples are fitted by biexponential kinetics function (Supplementary Note 1, Supplementary Equation 1) in which two decay components are derived (insets in Fig. 4b) (*τ*_1_ is originated from the nonradiative recombination of charge carriers in the defect states of ZnIn_2_S_4_, whereas the longer lifetime component of *τ*_2_ is caused by the recombination of free excitons in the ZnIn_2_S_4_). For ZnIn_2_S_4_/MoSe_2_, the emission lifetimes of both components (*τ*_1_=0.31 ns, *τ*_2_=0.38 ns) are shorter than that of the corresponding bulk ZnIn_2_S_4_ counterpart (*τ*_1_=0.73 ns, *τ*_2_=3.77 ns). The average emission lifetime (calculated from Supplementary Note 1, Supplementary Equation 2), which reflects the overall emission decay behaviour of sample, has also displayed an obvious decrease for ZnIn_2_S_4_/MoSe_2_ (2.43 ns) as compared with that of bulk ZnIn_2_S_4_ (3.78 ns). Meanwhile, the steady-state PL spectra in Fig. 4d show obvious PL quenching of hetero-layered ZnIn_2_S_4_/MoSe_2_ hybrid. The corresponding observations of PL quenching and lifetime reduction suggest the establishment of an electron transfer channel from ZnIn_2_S_4_ to MoSe_2_ in a nonradiative quenching pathway (Fig. 4c)^54^,^55^. Accordingly, this leads to efficient interfacial charge transfer and suppression of photoexcited charge recombination in the hetero-layered ZnIn_2_S_4_/MoSe_2_ structure.

Furthermore, the electrochemical impedance spectrum of ZnIn_2_S_4_/MoSe_2_ (Fig. 4e) shows a smaller semicircular in the Nyquist plot than that of bare ZnIn_2_S_4_ nanosheets, indicating a lower charge-transfer resistance in the hybrid composite that warrants efficient transportation and separation of charge carriers^56^,^57^,^58^,^59^. As shown in Fig. 4f, the ZnIn_2_S_4_/MoSe_2_ displays obvious transient photocurrent response under visible light irradiation. The current density is comparable to recently reported 2D-based photocatalyst systems (Supplementary Table 1). In addition, it is notable that the ZnIn_2_S_4_/MoSe_2_ displays ca. 22-fold photocurrent enhancement as compared with bulk ZnIn_2_S_4_ under the same experimental condition, and this is a marked improvement that suggests the structure and composition advantage of the hetero-layered ZnIn_2_S_4_/MoSe_2_ composite in promoting the separation and transportation of photogenerated charge carriers. Collectively, these commendable photo- and electrochemical properties support efficient electron–hole pair separation and high surface reaction rate of the ZnIn_2_S_4_/MoSe_2_ hetero-layered structure.

### Photocatalytic H_2_ production performance

Recent experimental results and theoretical predictions suggest that metal chalcogenides are a class of promising inexpensive, earth-abundant and visible responsive catalyst alternatives. However, nonoptimal interfacial contact and bulk metal chalcogenides structure limit catalytic activity owing to poor electronic coupling effect and low active sites exposure. Correspondingly, the constructed metal chalcogenide heterostructure that is thinned down to a few layers with incorporated electrostatic coupling is evaluated by photocatalytic H_2_ production. The photocatalytic performance provides a useful and explicit evaluation to assert the structural integrity and stability of the 2D hetero-layered structure.

**Figure 5:**
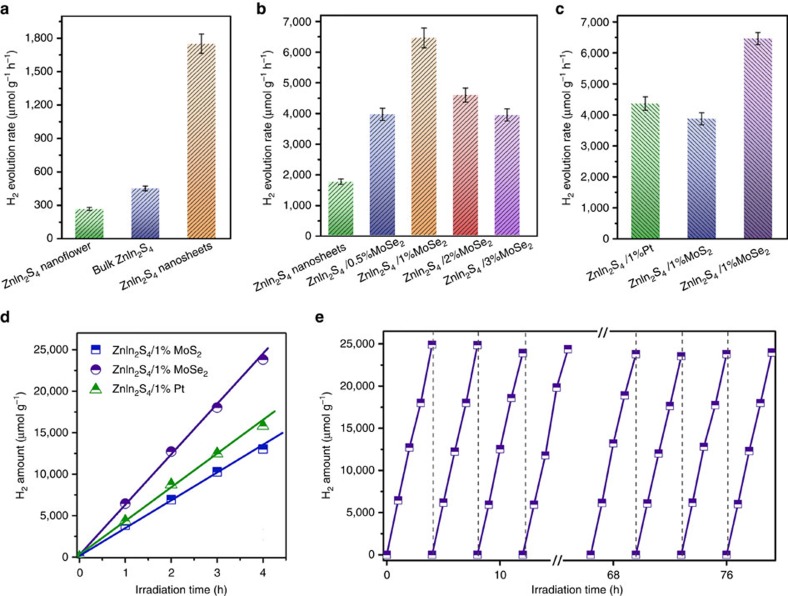
Photocatalytic H_2_ production performance. (**a**,**b**) Photocatalytic H_2_ evolution over ZnIn_2_S_4_ nanoflowers, bulk ZnIn_2_S_4_ and single-unit-cell ZnIn_2_S_4_ layers (**a**), and ZnIn_2_S_4_/MoSe_2_ composites with different weight ratios of MoSe_2_ (**b**). (**c**,**d**) Comparison of photocatalytic H_2_ evolution activities over ZnIn_2_S_4_/1%Pt, ZnIn_2_S_4_/1%MoS_2_ and ZnIn_2_S_4_/1%MoSe_2_. (**e**) Recycling photoactivity test of ZnIn_2_S_4_/1%MoSe_2_. Note that the error bars represent the photoactivity s.d. values calculated from triplicate experiments.

The photocatalytic activity, H_2_ generation of the ultrathin ZnIn_2_S_4_ layers and ZnIn_2_S_4_/MoSe_2_ hetero-layered nanohybrids was performed under visible light irradiation (*λ*>400 nm) using lactic acid as the hole scavenger. As shown in Fig. 5a, the single-unit-cell ZnIn_2_S_4_ layers show a H_2_ evolution rate of ∼1,748 μmol g^−1^ h^−1^ that is fourfold of the pristine bulk ZnIn_2_S_4_ (446 μmol g^−1^ h^−1^), indicating enhanced activity of the ultrathin layers. In addition, the photoactivity of the ultrathin ZnIn_2_S_4_ is also demonstrated to be much higher than the H_2_ generation rate of the hydrothermal synthesized ZnIn_2_S_4_ nanoflowers (Supplementary Fig. 40) of ca. 260 μmol g^−^ h^−1^. The augmented photoactivity of ZnIn_2_S_4_ nanosheets can be attributed to its unique 2D ultrathin structure that lowers charge-transfer resistance and shortens the diffusion pathway of charge carriers, thus favouring the fast and efficient separation of photogenerated charge carriers (Supplementary Figs 16 and 17).

Furthermore, significant improvement in H_2_ generation was established after coupling of MoSe_2_ layers to the ultrathin ZnIn_2_S_4_ nanosheets forming hetero-layered composites. The amount of H_2_ evolved increases with MoSe_2_ content up to 1% (Fig. 5b). The as-obtained ZnIn_2_S_4_/1%MoSe_2_ displays the highest H_2_ evolution rate of 6,454 μmol g^−1^ h^−1^, that is ∼15 and 4 times as high as that of bulk ZnIn_2_S_4_ and ZnIn_2_S_4_ nanosheets, respectively. Notably, the photoactivity is considerable higher than that of the reference photocatalysts, that is, ZnIn_2_S_4_/1%Pt (4,353 μmol g^−1^ h^−1^) and ZnIn_2_S_4_/1%MoS_2_ (3,860 μmol g^−1^ h^−1^), as shown in Fig. 5c,d. Pt and MoS_2_ are two exemplary co-catalysts that have been used in reported literature for photocatalytic H_2_ evolution. The result highlights the effectiveness of MoSe_2_ that surpasses the classic Pt and MoS_2_, as a co-catalyst in promoting photocatalytic H_2_ evolution^44^,^60^. Importantly, the hetero-layered ZnIn_2_S_4_/MoSe_2_ displays good stability. The cycling test over the optimal ZnIn_2_S_4_/1%MoSe_2_ (Fig. 5e) shows negligible photoactivity loss after 20 consecutive cycles with accumulatively 80 h under visible light irradiation. Moreover, after storing in ambient conditions for 3 months, the ZnIn_2_S_4_/1%MoSe_2_ retains a high photoactivity as that of the fresh sample (Supplementary Fig. 41). The high photoreactivity and stability of the 2D hetero-layer ZnIn_2_S_4_/1%MoSe_2_ hybrid provide direct evidence of the large exposed active surface and strong electronic coupling between the interlayers.

Besides, photocatalytic H_2_ activities of ultrathin CdIn_2_S_4_ and In_2_S_3_ layers also exceed that of their bulk counterparts (Supplementary Fig. 42). Self-assembly construction of 2D hetero-layer hybrids, that is, CdIn_2_S_4_/MoSe_2_ and In_2_S_3_/MoSe_2_, also verifies the enhanced photoactivity and stability. Essentially, the coupling of ultrathin CdIn_2_S_4_ and In_2_S_3_ with a few layers of MoSe_2_ co-catalyst has enhanced the H_2_ evolution activity (Supplementary Fig. 43). The rates of H_2_ evolved over the optimal CdIn_2_S_4_/1%MoSe_2_ and In_2_S_3_/1%MoSe_2_ are ∼9- and 10-fold of bare CdIn_2_S_4_ and In_2_S_3_ nanosheets, respectively. In addition, the photoactivities of the resulted metal sulfide/MoSe_2_ (CdIn_2_S_4_/1%MoSe_2_ and In_2_S_3_/1%MoSe_2_) are also much higher than those of the reference photocatalysts, that is, metal sulfide/Pt and metal sulfide/MoS_2_ (Supplementary Fig. 44). Furthermore, the cycling tests of the optimal CdIn_2_S_4_/1%MoSe_2_ and In_2_S_3_/1%MoSe_2_ show negligible photoactivity degradation after 20 consecutive cycles with accumulatively 80 h under visible light irradiation (Supplementary Fig. 45).

## Discussion

Although prior literature has already reported exfoliated 2D layered metal chalcogenides commonly induced by intercalator osmotic swelling, self-surface charge exfoliation into single-unit-cell thick layer structure in pure water is unprecedented. Successive artificially coupled hetero-layered structure with ultrathin and intimate interface characteristics, guided by unconstraint electrostatic coordination of a dissimilar metal chalcogenide, is demonstrated in this work. In contrast, thin-film epitaxial growth is strongly influenced by the surface of the substrate and degree of lattice matching. This limits the production yield and imposes a high cost of 2D hetero-layer metal chalcogenide for practical implementation. To test the hypothesis of the satisfactorily high quality of the as-prepared 2D hetero-layer, surface and interfacial-dominated photocatalysis is used as an ideal testbed for reliability verification. Diverse 2D ultrathin metal sulfide/MoSe_2_ hetero-layered materials reveal outstanding visible-light photoreactivity and efficient charge transfer exceeding that of metal sulfide/Pt and metal sulfide/MoS_2_ reference photocatalysts. More remarkably, the ultrathin hetero-layer structures demonstrate highly stable contact interface promising for long-term cycling and storage purposes.

In summary, we have developed a scalable method to artificially coordinate ultrathin metal chalcogenide hetero-layer structure via a combination of pristine self-surface charge exfoliation and electrostatic coupling of dissimilar layers. This generic approach to the preparation of 2D hetero-layered hybrids is attractive as it allows selection of individual constituent materials and thickness control, opening up the possibility of ‘design-and-build' 2D layered heterojunction for large-scale theoretical exploration and practical applications.

## Methods

### Materials

Zinc acetate dehydrate (Zn(CH_3_COO)_2_·2H_2_O, ≥98%), sodium molybdate dehydrate (Na_2_MoO_4_·2H_2_O, ≥99%) and hydrazine hydrate (N_2_H_4_·H_2_O, 99.99%) solution were obtained from Sigma-Aldrich. Indium chloride (InCl_3_, 99.995%), L(+) lactic acid (90%) and selenium (99.5+%) were obtained from ACROS Organics. Thioacetamide (C_2_H_5_NS, >98%) was obtained from TCI. All of the reagents were used as received without further purification. The DI water used in the catalyst preparation was from local sources.

### Synthesis of single-unit-cell ZnIn_2_S_4_ layers

Single-unit-cell ZnIn_2_S_4_ layers were fabricated by a facile low-temperature refluxing method followed by a moderate exfoliation. In detail, 1.5 mmol of Zn(CH_3_COO)_2_·2H_2_O and 3 mmol of InCl_3_ were added into 250 ml DI water and stirred for 30 min. Subsequently, an excess amount of thioacetamide (TAA, 8 mmol) was added into the above solution and stirred for another 30 min. The solution was then heated to 95 °C and maintained at that temperature for 5 h under vigorous stirring. The resulted precipitation was collected by centrifugation, rinsed with water for 2 times and re-dispersed into 200 ml DI water. The dispersion was sonicated continuously for 30 min and then centrifuged at 6,000 r.p.m. for 5 min to remove aggregates. After that, the colloidal single-unit-cell ZnIn_2_S_4_ layers were obtained. For each set of experimental synthesis, ∼0.6 g of ZnIn_2_S_4_ can be obtained. The comparative sample of ZnIn_2_S_4_-S was synthesized via the same procedure except that a stoichiometric amount of TAA (6 mmol) was added during the synthesis process.

### Synthesis of hetero-layered ZnIn_2_S_4_/MoSe_2_ structure

MoSe_2_ was synthesized via a one-step hydrothermal method^44^. The surface modification of MoSe_2_ was carried out as follows: 50 mg MoSe_2_ was dispersed in 100 ml ethanol and sonicated continuously for 1 h. Then, 0.25 ml of APTES was added into the above MoSe_2_ dispersion. The mixture was heated at 60 °C for 4 h under mild stirring. The resulted product was rinsed with ethanol for 3 times and redispersed in 100 ml DI water with the aid of ultrasonication for 1 h. After that, the dispersion was centrifuged at 6,000 r.p.m. for 5 min to remove aggregates. Then, the colloid APTES-modified MoSe_2_ with positive surface charge was obtained. The 2D hetero-layer composite can be built up by dipping of MoSe_2_ nanosheet suspension into colloidal ZnIn_2_S_4_ of constraint supply or controlled amount of dilute solution. In brief, the APTES-modified MoSe_2_ was added dropwise into the negatively charged ZnIn_2_S_4_ colloid slowly. Driving by the strong electrostatic attractive interaction, the ultrathin ZnIn_2_S_4_ nanosheets can self-assemble with the MoSe_2_ layers, forming intimately integrated ZnIn_2_S_4_/MoSe_2_ hetero-layer structure.

### Synthesis of ultrathin CdIn_2_S_4_ and In_2_S_3_ layers

Ultrathin CdIn_2_S_4_ layers were fabricated via the similar facile low-temperature refluxing method followed by moderate exfoliation. In detail, 1.5 mmol of Cd(CH_3_COO)_2_·2H_2_O and 3 mmol of InCl_3_ were added into 250 ml DI water and stirred for 30 min. Subsequently, an excess amount of thioacetamide (TAA, 8 mmol) was added into the above solution and stirred for another 30 min. The solution was then heated to 100 °C and maintained at that temperature for 12 h under vigorous stirring. The resulted precipitation was collected by centrifugation, rinsed with water for 2 times and redispersed into 200 ml DI water. The dispersion was sonicated continuously for 30 min and then centrifuged at 6,000 r.p.m. for 5 min to remove aggregates. After that, the colloidal ultrathin ZnIn_2_S_4_ layers were obtained. Ultrathin In_2_S_3_ layers were synthesized via the same method as that of synthesizing ZnIn_2_S_4_ layers without the addition of Zn(CH_3_COO)_2_·2H_2_O precursor.

### Synthesis of hetero-layered CdIn_2_S_4_/MoSe_2_ and In_2_S_3_/MoSe_2_

The hetero-layered CdIn_2_S_4_/MoSe_2_ and In_2_S_3_/MoSe_2_ were synthesized following the same procedure as that of preparing of ZnIn_2_S_4_/MoSe_2_.

### Characterization

The XRD patterns of the samples were collected on a Philips X-ray diffractometer with Cu Kα radiation (*λ*=1.541 Å). UV–vis absorption spectra were recorded on a Shimadzu UV-3600 UV–vis spectrophotometer. XPS measurement was performed on a Thermo Scientific ESCA Lab 250 spectrometer that consists of a monochromatic Al Kα as the X-ray source, a hemispherical analyser and sample stage with multiaxial adjustability to obtain the surface composition of the samples. All of the binding energies were calibrated by the C 1 s peak at 284.6 eV. Zeta-potential (ξ) measurements of the samples were determined by dynamic light scattering analysis (Zeta sizer 3000HSA) at a room temperature of 25 °C. SEM images were taken on a JEOL JSM-7001F field emission scanning electron microscope. HRTEM images, EDX and elemental mapping images were obtained on a JEOL JEM-2100 electron microscope. The steady-state PL spectra were recorded on a Shimazu RF-5301PC under the excitation of 400 nm. Tapping-mode AFM measurement was performed on a commercial SPM instrument (MPF-3D, Asylum Research, CA, USA).

Time-resolved photoluminescence measurement was performed under excitation of 400 nm fs pulses. The excitation source is a mode-locked Ti:sapphire laser (Chameleon Ultra II, Coherent) working with repetition rate of 80 MHz and pulse duration of 140 fs. The second harmonic generation of 700 nm output from the laser was employed to excite the samples. The photoluminescence of the samples was collected and detected by a photon-counting photomultiplier (PMA, Picoquant). The emission centred at 495 nm was selected by a monochrometer (SpectroPro 2300i, Princeton Instrument). The PL decay dynamics were achieved by a time-correlated single photon counting module (TCSPC Picoharp 300, Picoquant).

Photoelectrochemical measurements were performed in a conventional three-electrode quartz cell. A Pt plate was used as counter electrode, and Ag/AgCl electrode/saturated calomel electrode were used as reference electrode, whereas the working electrode was prepared on FTO conductor glass. The sample powder (3 mg) was ultrasonicated in 0.5 ml of *N*,*N*-dimethylformamide (supplied by Sigma-Aldrich) to disperse it evenly to get a slurry. The slurry was spread onto FTO glass with the area of 1 cm^2^. After air drying, the working electrode was further dried at 90 °C for 2 h to improve adhesion. The electrolyte was 0.2 M aqueous Na_2_SO_4_ solution (pH=6.8). Linear sweep voltammetry curves were performed in a mixed solution of 10% (v/v) lactic acid and 0.2 M aqueous Na_2_SO_4_ solution.

### Photocatalytic H_2_ evolution measurements

With the aid of ultrasonication, 5 mg of photocatalyst, 9 ml DI water and 1 ml lactic acid were mixed in a 25 ml quartz cylindrical reaction cell to form a homogeneous suspension. Then, the reactor was purged with argon gas for 10 min before illumination with a 300 W xenon arc lamp (*λ*>400 nm). The evolved H_2_ was analysed using an online gas chromatograph (GC-2014AT, Shimadzu Co., Japan) equipped with a thermal conductivity detector.

The recycling test of catalytic H_2_ evolution over the as-prepared photocatalyst was performed as follows. After the reaction of the first run under visible light irradiation, the suspension was purged with argon gas for 10 min. The process was carried out for four more cycles. After every five cycles, the photocatalyst was centrifuged and mixed with fresh 9 ml DI water and 1 ml lactic acid for continuous test.

### Data availability

The data that support the findings of this study are available from the corresponding author on request.

## Additional information

**How to cite this article:** Yang, M.-Q. *et al*. Self-surface charge exfoliation and electrostatically coordinated 2D hetero-layered hybrids. *Nat. Commun.* **8,** 14224 doi: 10.1038/ncomms14224 (2017).

**Publisher's note**: Springer Nature remains neutral with regard to jurisdictional claims in published maps and institutional affiliations.

## Supplementary Material

Supplementary Information Supplementary Figures, Supplementary Table, Supplementary Note and Supplementary References.
