# Supplementary material for: Self-surface charge exfoliation and electrostatically coordinated 2D hetero-layered hybrids
Source: Nat Commun. 2017 Feb 1;8:14224. doi: 10.1038/ncomms14224 (PMC5296640; doi:10.1038/ncomms14224)
Supplement: Supplementary Information — Supplementary Figures, Supplementary Table, Supplementary Note and Supplementary References. [file ncomms14224-s1.pdf]

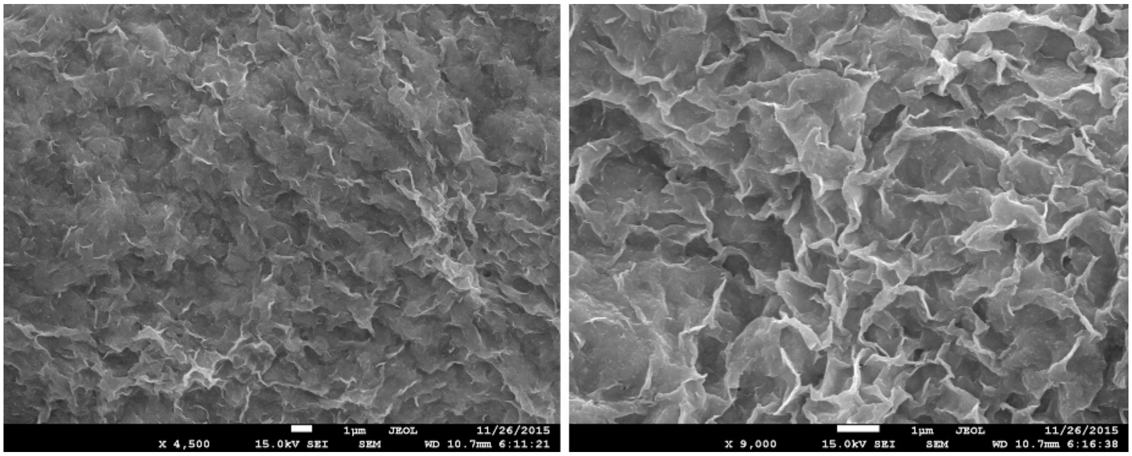

Supplementary Figure 1. Additional scanning electron microscopy (SEM) images of bulk $ZnIn_2S_4$.

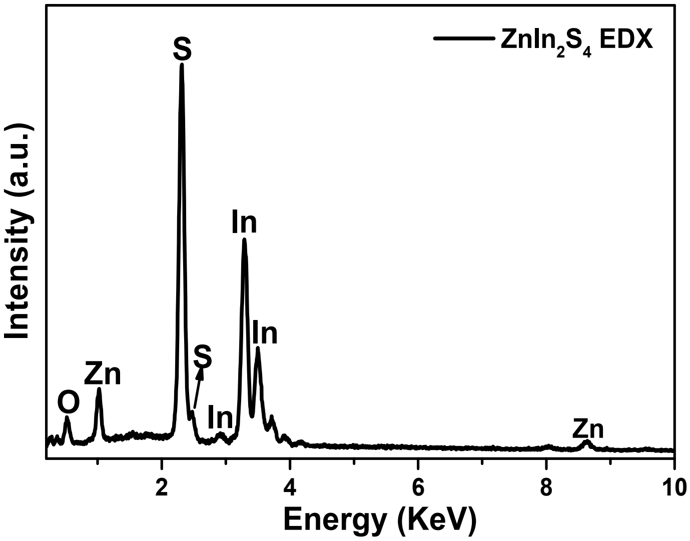

Supplementary Figure 2. Energy-dispersive X-ray (EDX) spectrum of $ZnIn_2S_4$.

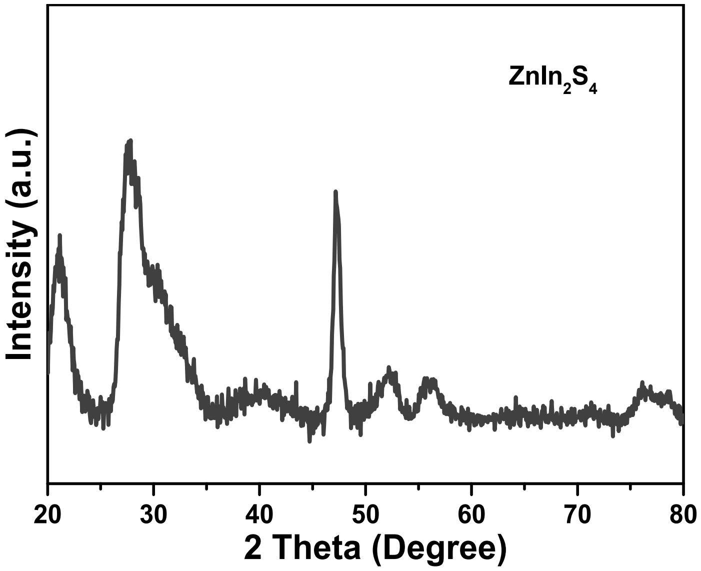

Supplementary Figure 3. X-ray diffraction (XRD) pattern of $ZnIn_2S_4$.

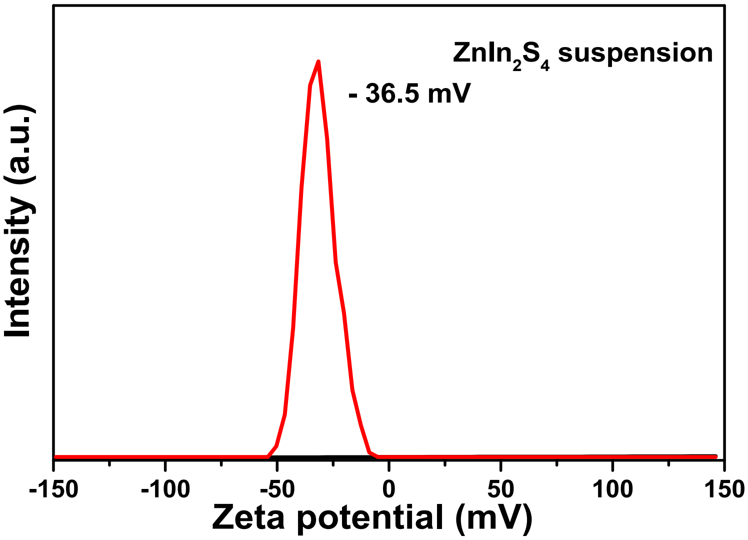

Supplementary Figure 4. Zeta potential of $ZnIn_2S_4$ suspension dispersed in deionized water.

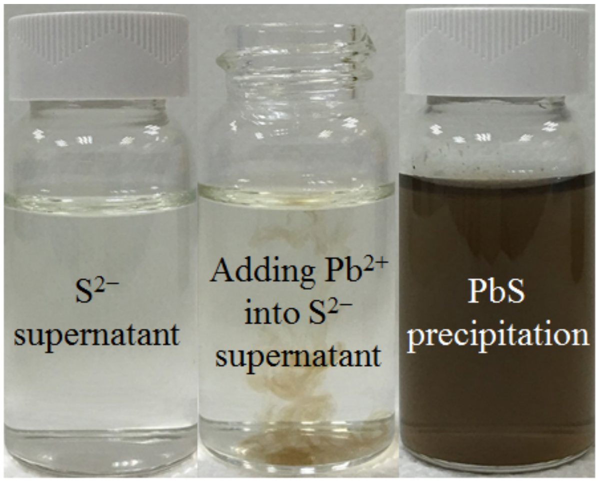

Supplementary Figure 5. Photographs of S^{2-} supernatant with the addition of Pb^{2+}. The excess amount of S^{2-} precursor is validated by the addition of Pb^{2+} into the supernatant of $ZnIn_2S_4$ that leads to precipitation of PbS nanoparticles.

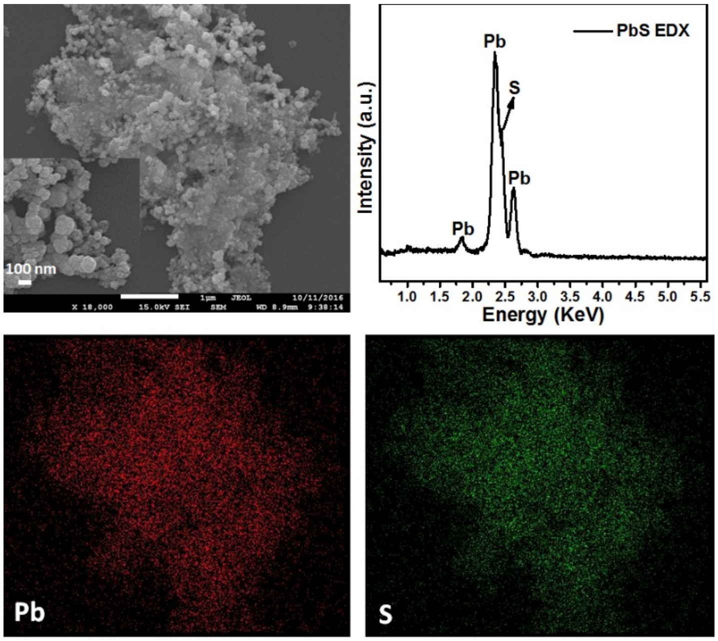

Supplementary Figure 6. SEM image, Energy-dispersive X-ray (EDX) spectrum and elemental mapping analysis of PbS precipitation. The EDX mapping confirms the elemental composition and distribution of PbS nanoparticles, which have been successfully precipitated from excess S^{2-} supernatant.

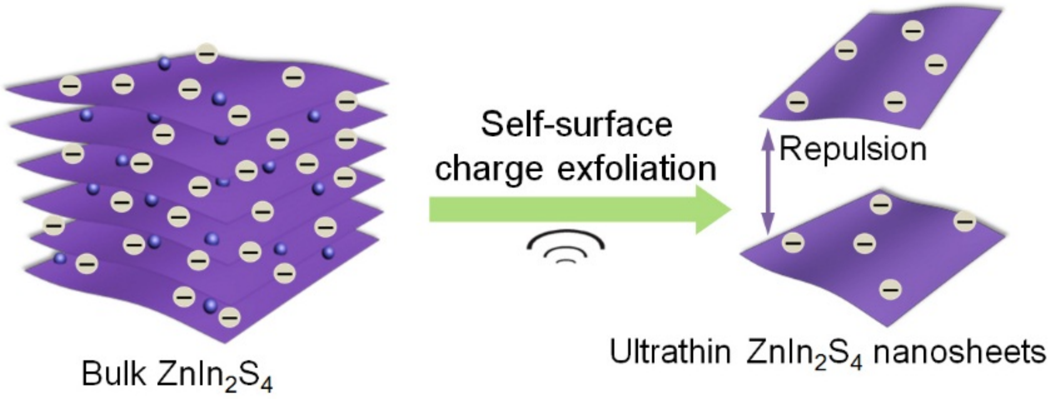

Supplementary Figure 7. Schematic illustration of the self-surface charge exfoliation of $ZnIn_2S_4$ sample synthesized from the addition of excess amount of thioacetamide.

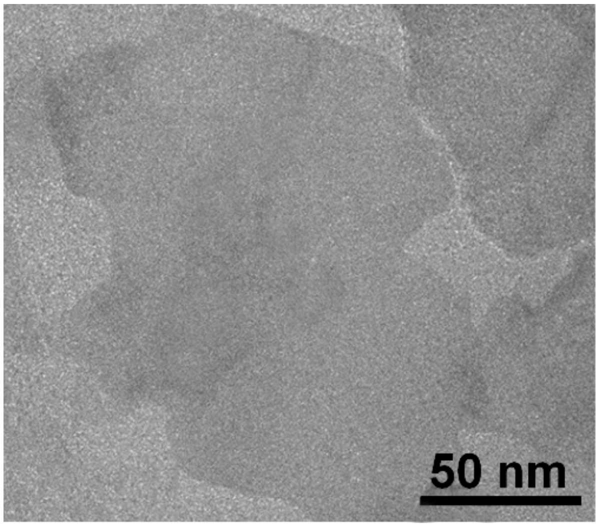

Supplementary Figure 8. Additional transmission electron microscopy (TEM) image of ultrathin $ZnIn_2S_4$ nanosheets.

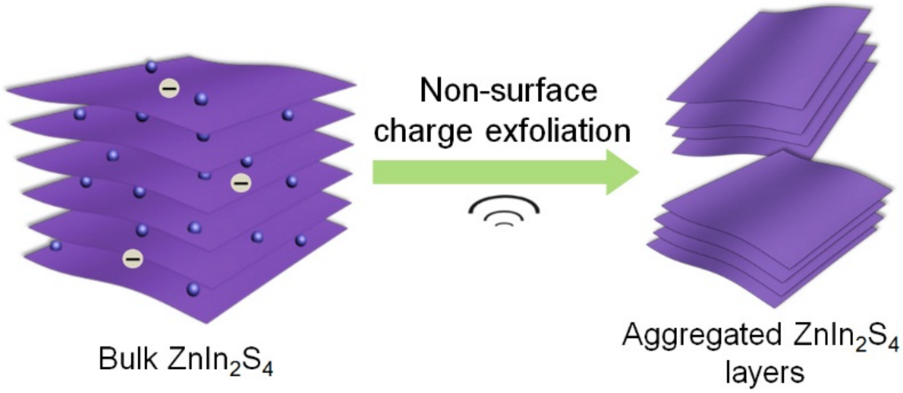

Supplementary Figure 9. Schematic illustration of the non-surface charge exfoliation of $ZnIn_2S_4$-S sample synthesized from the addition of stoichiometric amount of thioacetamide.

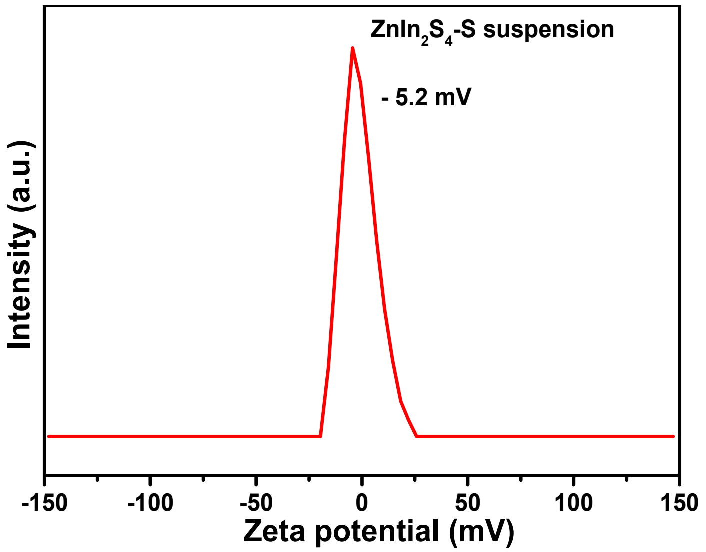

Supplementary Figure 10. Zeta potential of $ZnIn_2S_4$-S suspension dispersed in deionized water.

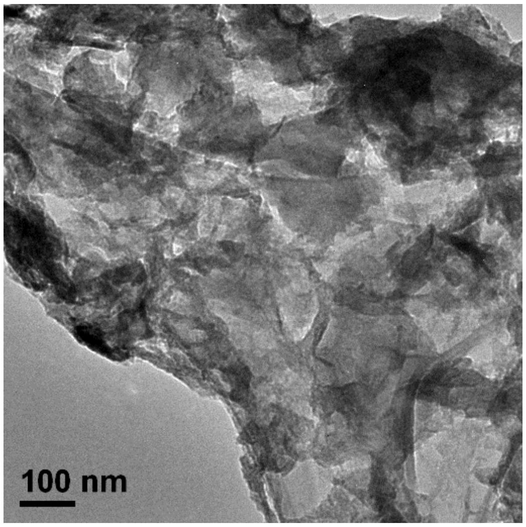

Supplementary Figure 11. Transmission electron microscopy (TEM) image of $ZnIn_2S_4$-S sample.

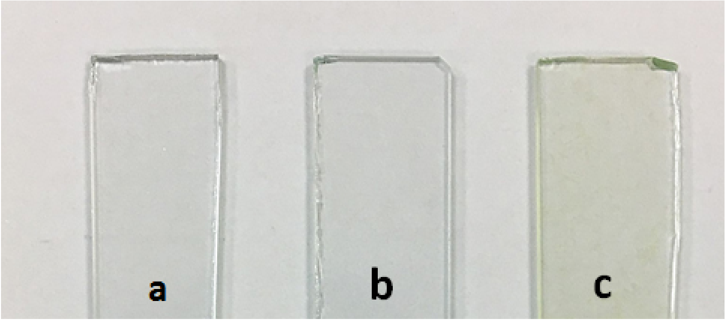

Supplementary Figure 12. Photographs of 3-aminopropyl-triethoxysilane (APTES)-modified glass substrate: (a) blank, (b) immersed in $ZnIn_2S_4$-S suspension with zeta potential of -5.2 mV, and (c) immersed in $ZnIn_2S_4$ colloid with zeta potential of -36.5 mV.

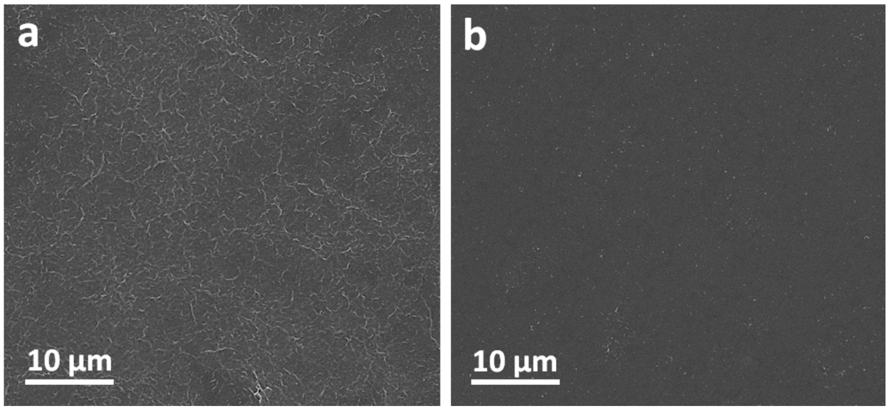

Supplementary Figure 13. SEM images of 3-aminopropyl-triethoxysilane (APTES)-modified glass substrate after immersing in $ZnIn_2S_4$ solution with (a) zeta potential of -36.5 mV, and (b) zeta potential of -5.2 mV.

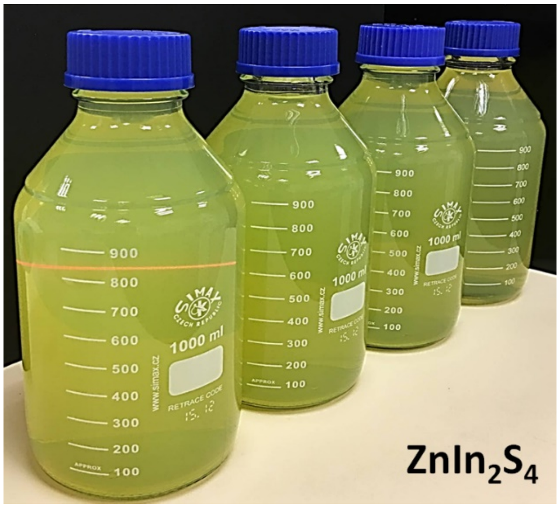

Supplementary Figure 14. Large-scale exfoliation of the ultrathin $ZnIn_2S_4$ layers (Note: Glass bottles are each 1000 mL).

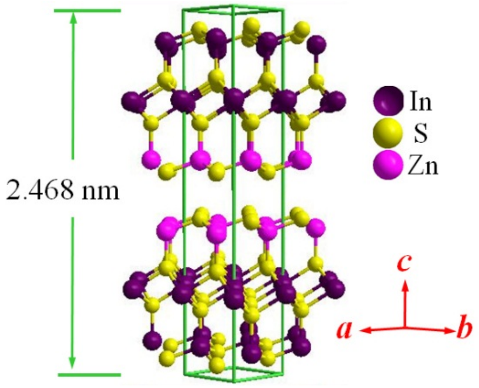

Supplementary Figure 15. The structural model of the layered $ZnIn_2S_4$.

Cell Parameters: $a = b = 3.85$ Å, $c = 24.68$ Å

In a unit cell of $ZnIn_2S_4$, alternating layers of the cations and anions are stacked perpendicular to the [001] axis.

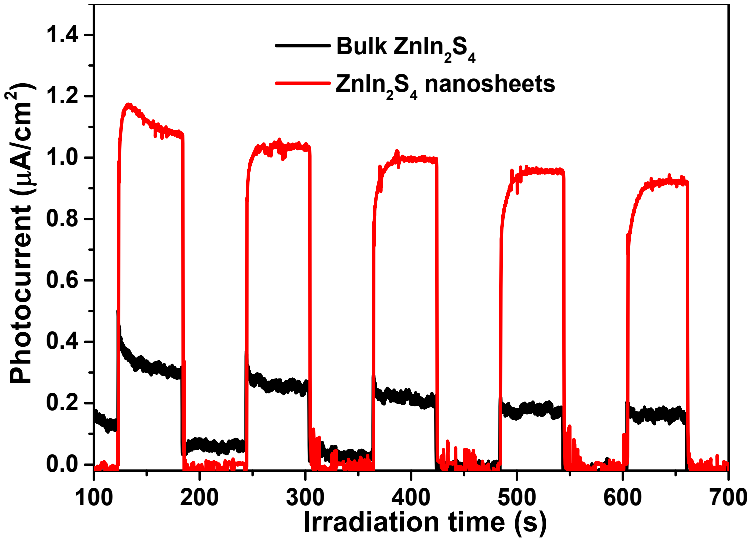

Supplementary Figure 16. Photocurrent spectra of bulk $ZnIn_2S_4$ and single-unit-cell $ZnIn_2S_4$ layers.

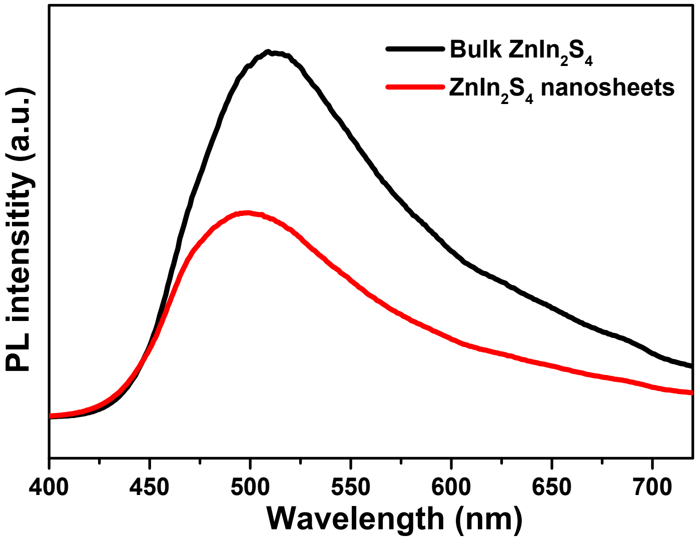

Supplementary Figure 17. Photoluminescence (PL) spectra of bulk $ZnIn_2S_4$ and single-unit-cell $ZnIn_2S_4$ layers.

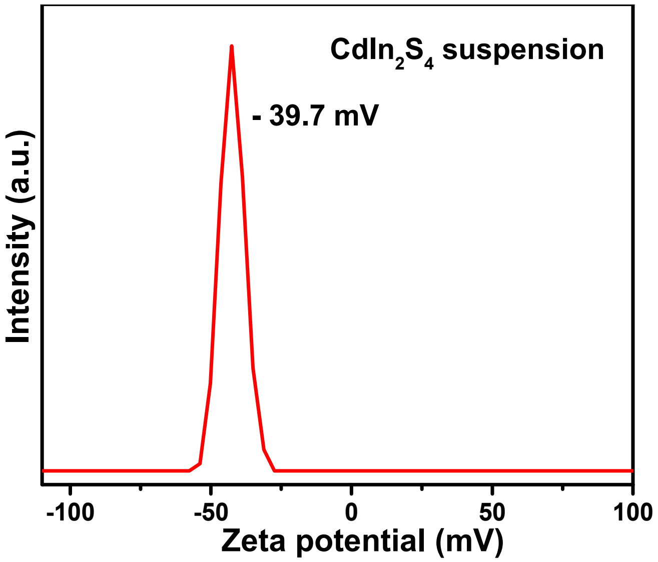

Supplementary Figure 18. Zeta potential of $CdIn_2S_4$ suspension dispersed in deionized water. The Zeta potential analysis of the $CdIn_2S_4$ dispersion in deionized water shows a strong negative charge with a zeta potential value of -39.7 mV, which promotes self-surface charge exfoliation of $CdIn_2S_4$ via electrostatic repulsion between the nanosheets.

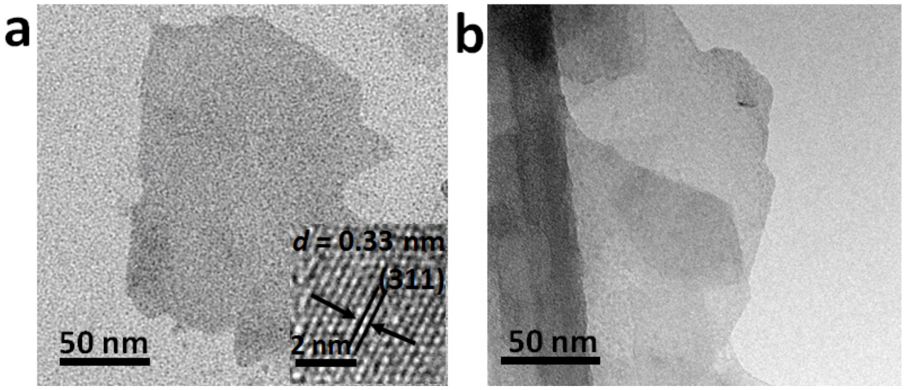

Supplementary Figure 19. Transmission electron microscopy (TEM) images of ultrathin $CdIn_2S_4$ nanosheets. $CdIn_2S_4$ is exfoliated into ultrathin nanosheets without any surfactant or intercalator under moderate ultrasonication. TEM images confirm the 2D sheet structure of the exfoliated $CdIn_2S_4$. High-resolution TEM (HRTEM) image displays distinct lattice fringes of ca. 0.33 nm, corresponding to the (311) crystallographic plane of $CdIn_2S_4$.

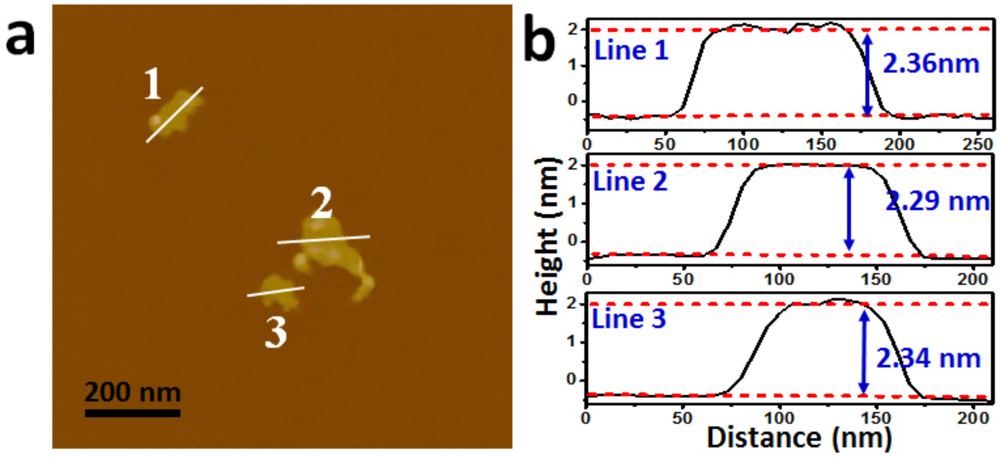

Supplementary Figure 20. Atomic Force Microscopy (AFM) image and corresponding height images of ultrathin $CdIn_2S_4$ nanosheets. The AFM image and height analysis reveal the thickness of the obtained $CdIn_2S_4$ is about 2.3 nm, which directly confirms the ultrathin nature of $CdIn_2S_4$ nanosheets.

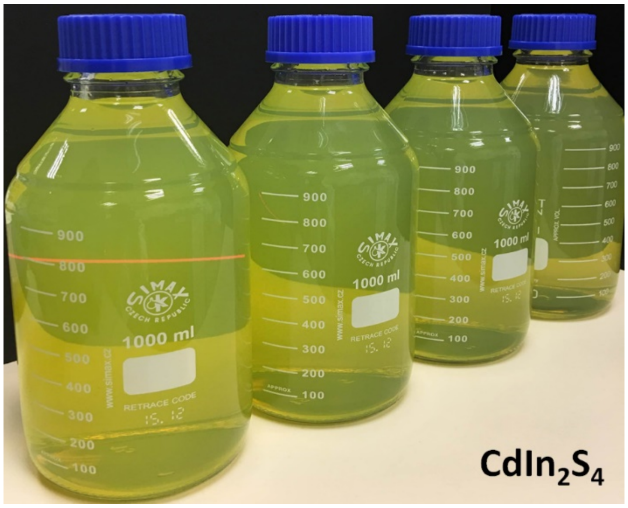

Supplementary Figure 21. Large-scale exfoliation of the ultrathin $CdIn_2S_4$ layers (Note: Glass bottles are each 1000 mL). Typical Tyndall effect observed for the as-exfoliated $CdIn_2S_4$ suspension implies the formation of large-scale freestanding and highly dispersed ultrathin $CdIn_2S_4$ layers.

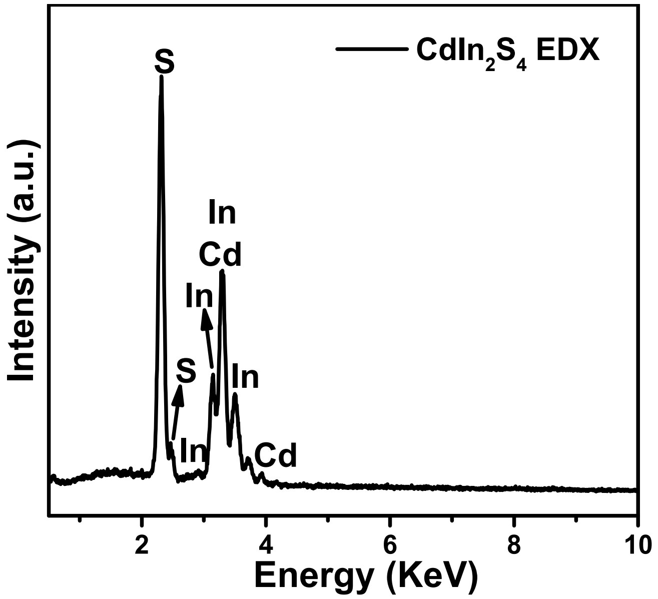

Supplementary Figure 22. Energy-dispersive X-ray (EDX) spectrum of $CdIn_2S_4$. The EDX spectrum confirms the elemental composition of $CdIn_2S_4$.

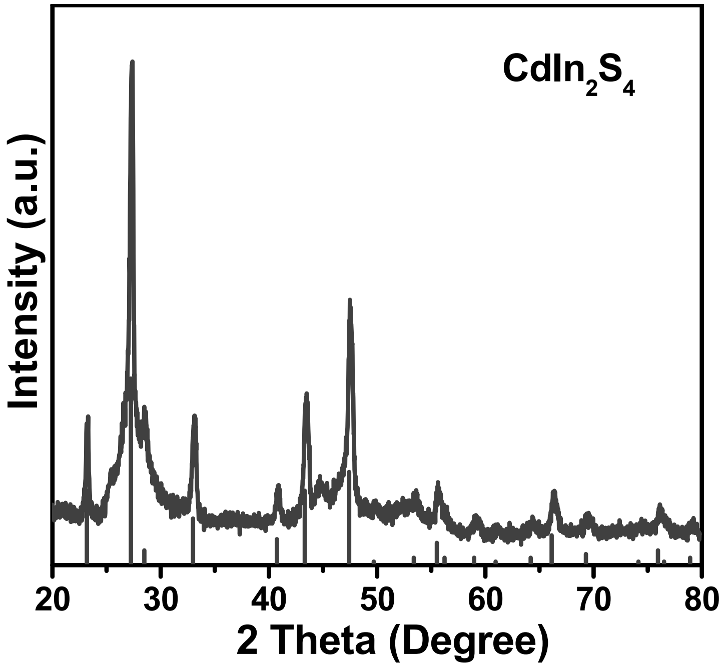

Supplementary Figure 23. X-ray diffraction (XRD) pattern of $CdIn_2S_4$. The XRD pattern shows the high purity of the as-synthesized $CdIn_2S_4$ with cubic phase structure (cell parameters of $a = b = c = 10.85$ Å, JCPDS No. 27-0060).

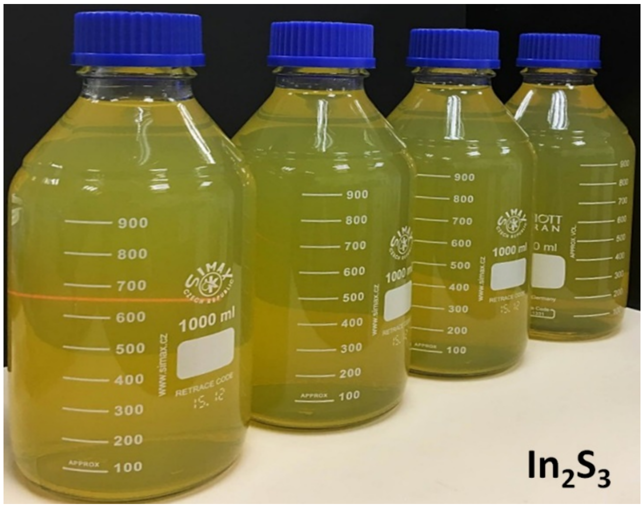

Supplementary Figure 24. Large-scale exfoliation of the ultrathin In_2S_3 layers (Note: Glass bottles are each 1000 mL). Typical Tyndall effect observed for the as-exfoliated In_2S_3 suspension implies the formation of large-scale freestanding and highly dispersed ultrathin In_2S_3 layers.

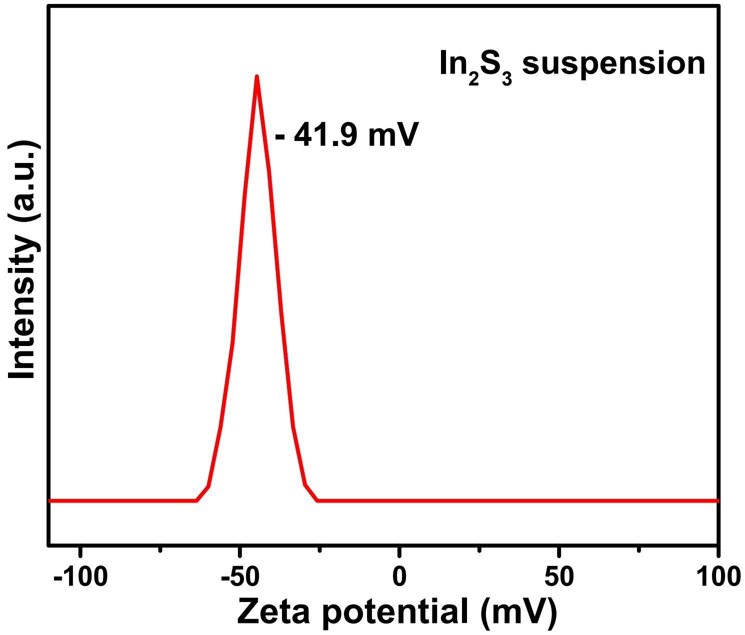

Supplementary Figure 25. Zeta potential of In_2S_3 suspension dispersed in deionized water. The Zeta potential measurement displays a strong negatively charged surface (zeta potential of -41.9 mV) of In_2S_3, which facilitates the exfoliation of In_2S_3 nanosheets via electrostatic repulsion.

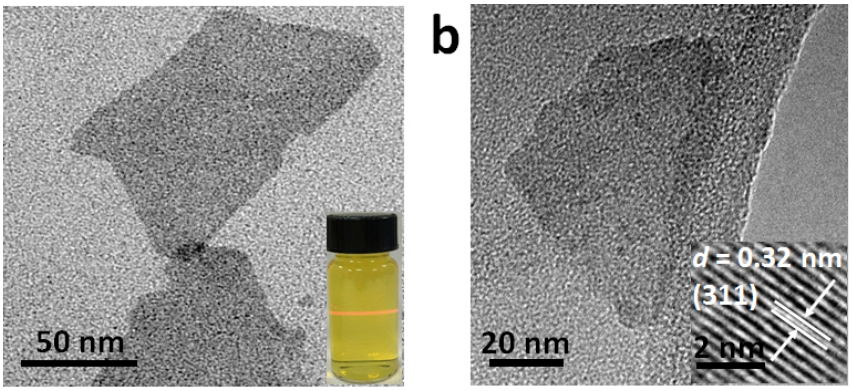

Supplementary Figure 26. Transmission electron microscopy (TEM) images of ultrathin In_2S_3 nanosheets. In_2S_3 is exfoliated into ultrathin nanosheets under moderate ultrasonication in the absence of surfactant or intercalator. TEM images show the 2D sheet structure of the exfoliated In_2S_3. High-resolution TEM (HRTEM) image displays distinct lattice fringes (ca. 0.32 nm) of (311) crystallographic plane of In_2S_3.

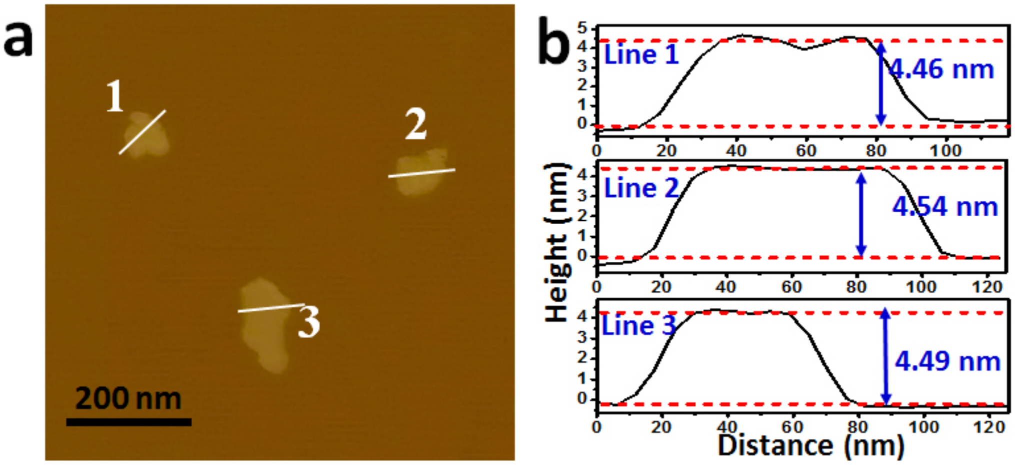

Supplementary Figure 27. Atomic Force Microscopy (AFM) image and corresponding height images of ultrathin In_2S_3 nanosheets. The AFM image and height analysis reveal the thickness of the obtained In_2S_3 is about 4.5 nm, which shows the ultrathin nature of the exfoliated In_2S_3 nanosheets.

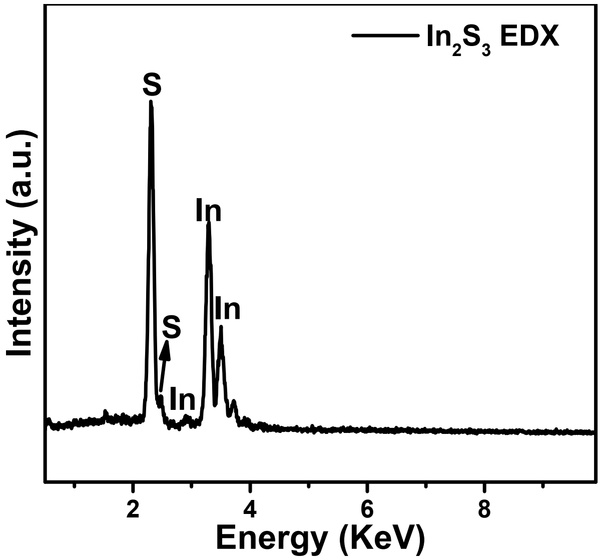

Supplementary Figure 28. Energy-dispersive X-ray (EDX) spectrum of In_2S_3. The EDX spectrum confirms the elemental composition of In_2S_3.

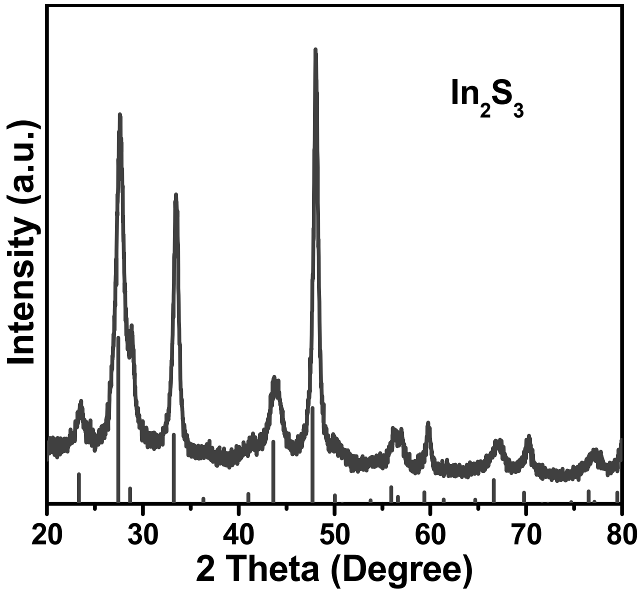

Supplementary Figure 29. X-ray diffraction (XRD) pattern of In_2S_3. The XRD peaks are well indexed to the cubic In_2S_3 (cell parameters of $a = b = c = 10.77$ Å, JCPDS No. 65-0459), indicating the high purity of the as-synthesized In_2S_3.

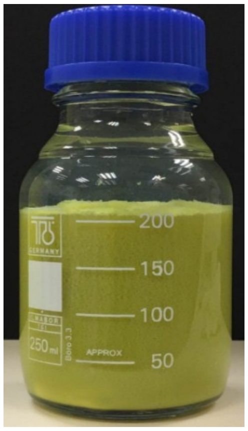

Supplementary Figure 30. Digital photograph of large quantity of hetero-layered $ZnIn_2S_4/MoSe_2$ hybrid composites (Note: Glass bottle is 250 mL).

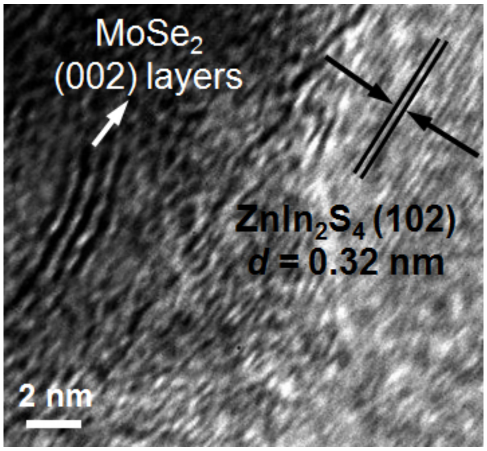

Supplementary Figure 31. Additional high-resolution transmission electron microscopy (HRTEM) image of $ZnIn_2S_4/MoSe_2$.

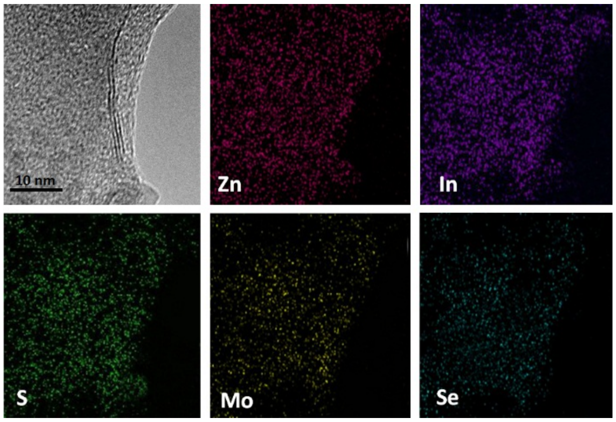

Supplementary Figure 32. TEM mapping analysis of $ZnIn_2S_4/MoSe_2$ hybrids.

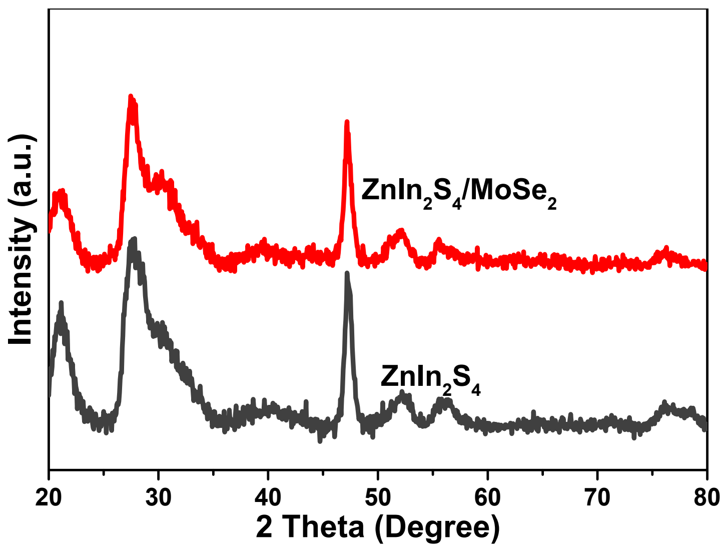

Supplementary Figure 33. X-ray diffraction (XRD) patterns of $ZnIn_2S_4$ and hetero-layer structured $ZnIn_2S_4/MoSe_2$.

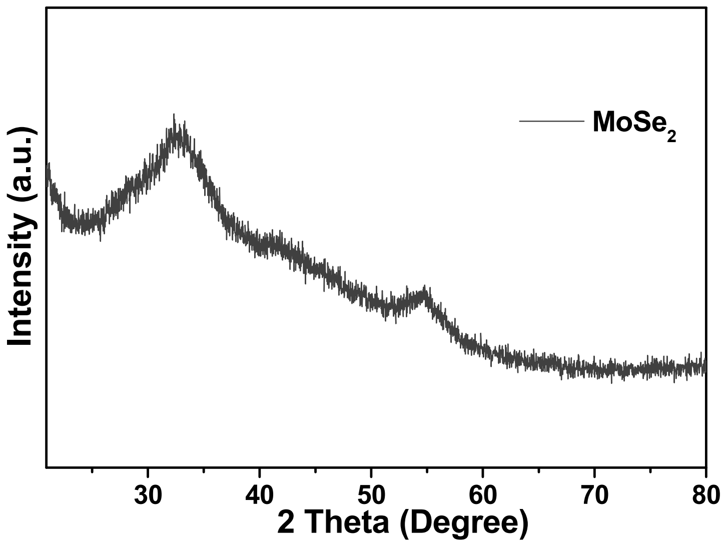

Supplementary Figure 34. X-ray diffraction (XRD) pattern of $MoSe_2$.

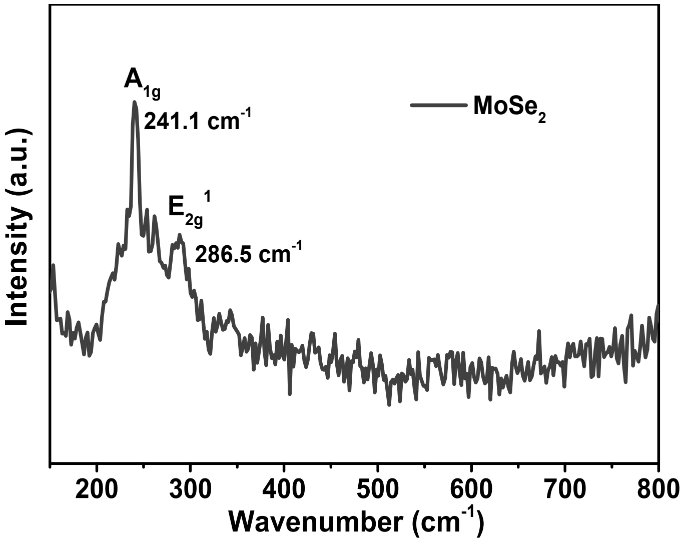

Supplementary Figure 35. Raman spectrum of $MoSe_2$.

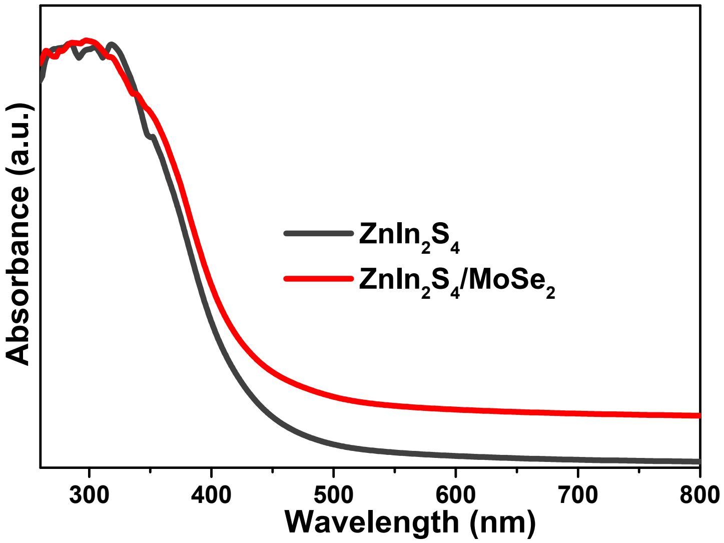

Supplementary Figure 36. UV-vis absorption spectra of $ZnIn_2S_4$ and hetero-layer structured $ZnIn_2S_4/MoSe_2$.

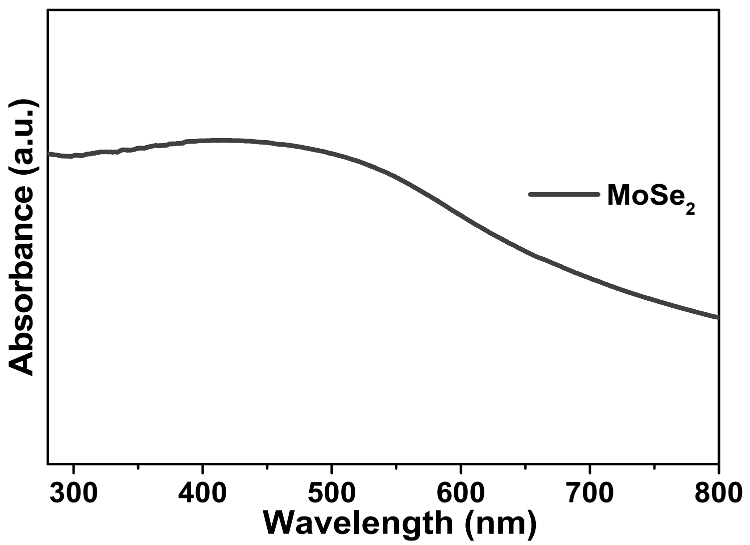

Supplementary Figure 37. UV-vis absorption spectrum of $MoSe_2$.

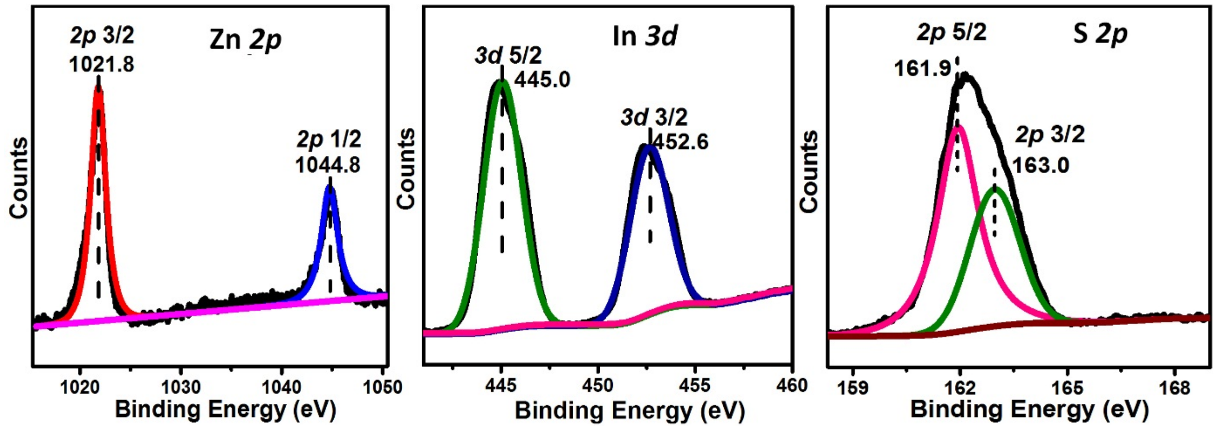

Supplementary Figure 38. High-resolution X-ray photoelectron spectroscopy (XPS) spectra of Zn *2p*, In *3d*, and S *2p* of hetero-layered $ZnIn_2S_4/MoSe_2$ composite.

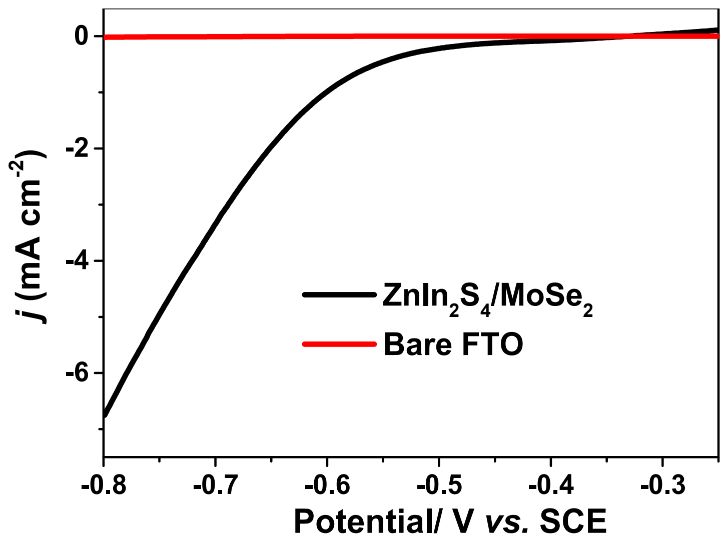

Supplementary Figure 39. Linear sweep voltammetry (LSV) curves of bare FTO and $ZnIn_2S_4/MoSe_2$.

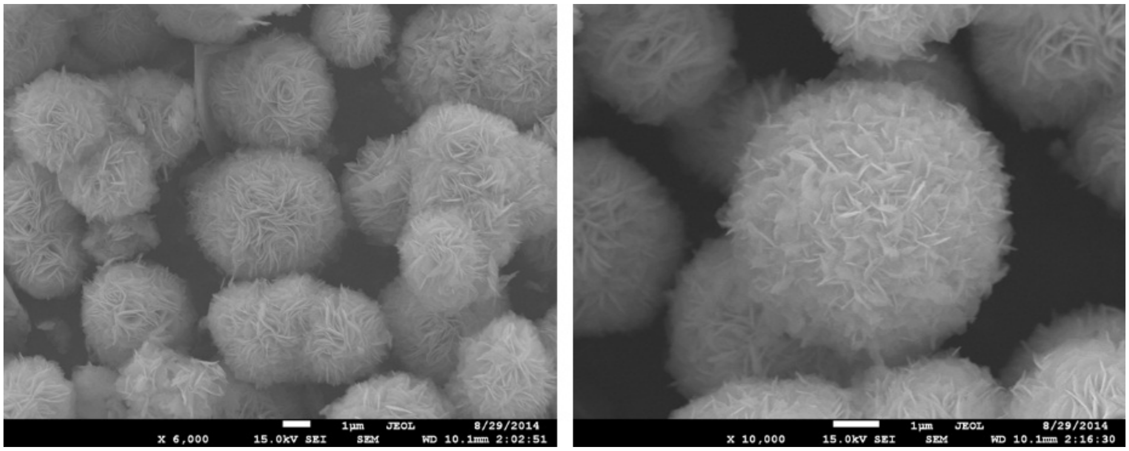

Supplementary Figure 40. Scanning electron microscopy (SEM) images of hydrothermal synthesized $ZnIn_2S_4$ nanoflowers.

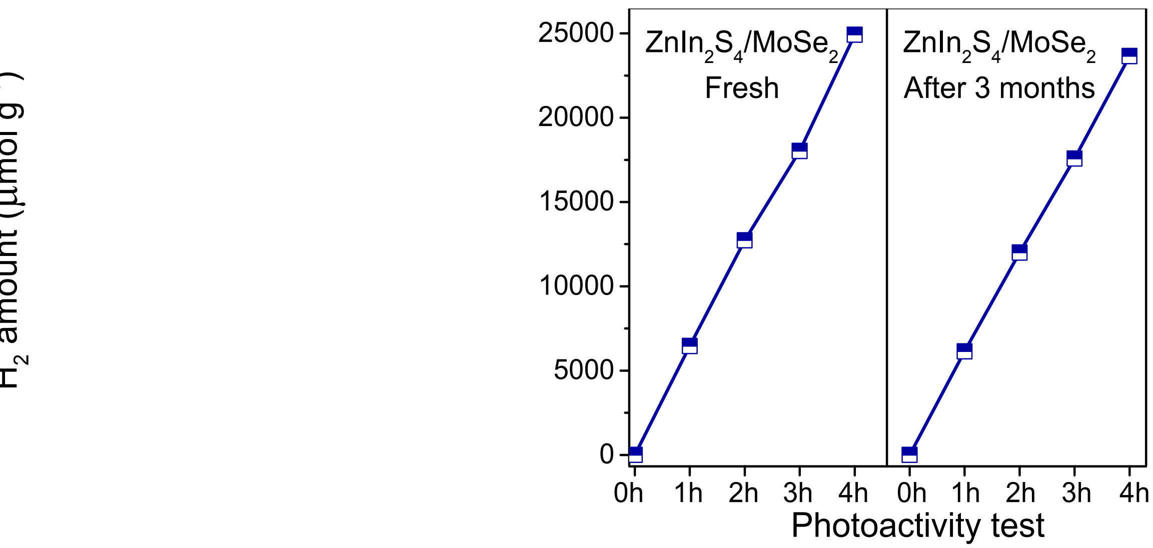

Supplementary Figure 41. Photoactivity test of fresh $ZnIn_2S_4$/1%$MoSe_2$ and after storing under ambient conditions for three months.

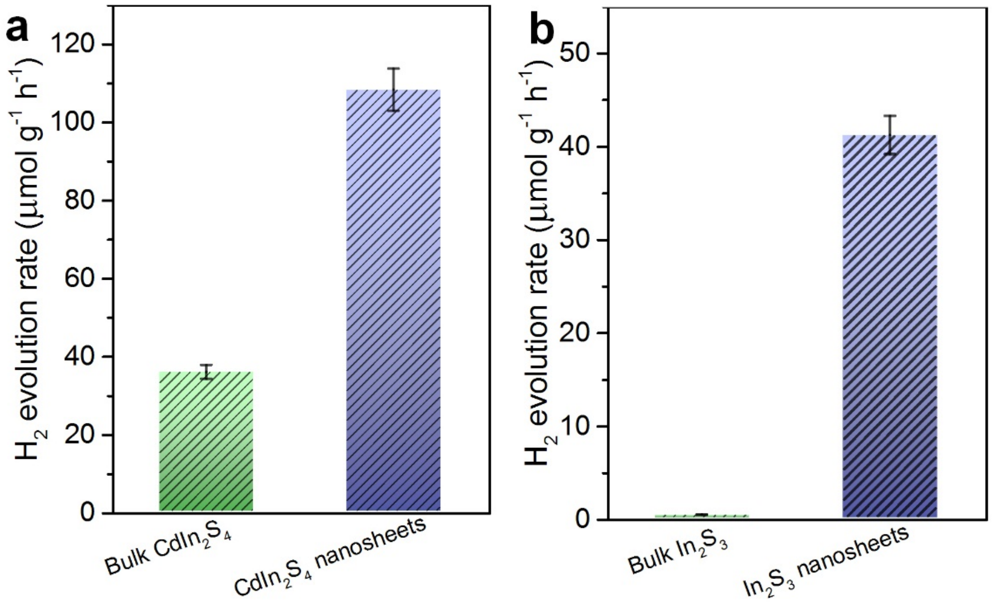

Supplementary Figure 42. Photocatalytic H_2 evolution over (a) bulk $CdIn_2S_4$ and ultrathin $CdIn_2S_4$ layers; (b) bulk In_2S_3 and ultrathin In_2S_3 layers. The photocatalytic H_2 production of ultrathin $CdIn_2S_4$ (108.2 μmol g^{-1} h^{-1}) and In_2S_3 (41.6 μmol g^{-1} h^{-1}) nanosheets both display higher H_2 evolution rate than their bulk counterparts of $CdIn_2S_4$ (35.7 μmol g^{-1} h^{-1}) and In_2S_3 (negligible). This indicates the enhanced activities of the exfoliated ultrathin metal sulphide layers. Note: the error bars represent the photoactivity standard deviations calculated from triplicate experiments.

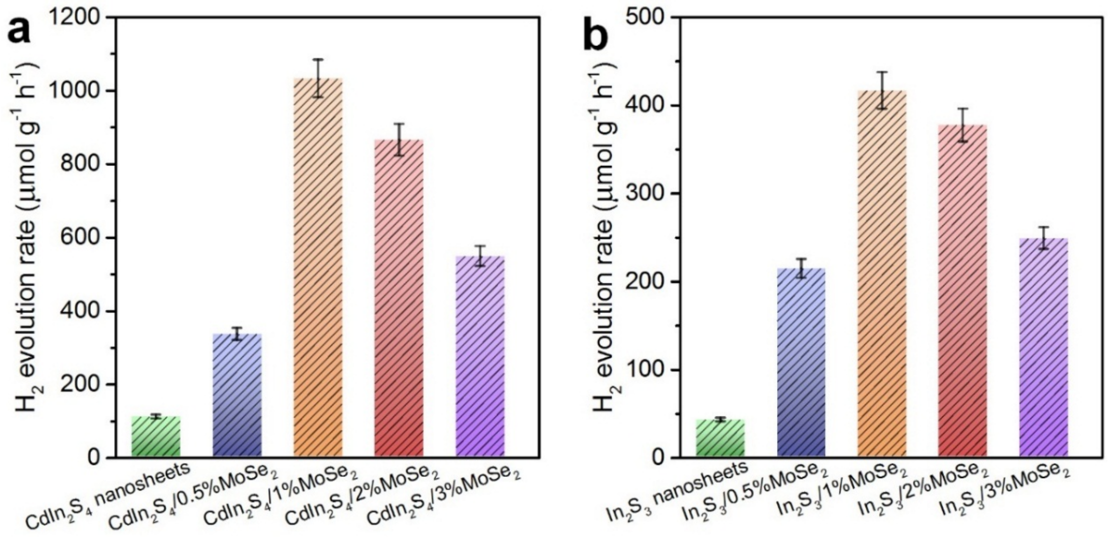

Supplementary Figure 43. The photoactivities of (a) $CdIn_2S_4/MoSe_2$ and (b) $In_2S_3/MoSe_2$ hetero-layered nanohybrids with different weight ratios of $MoSe_2$. Note: the error bars represent the photoactivity standard deviations calculated from triplicate experiments.

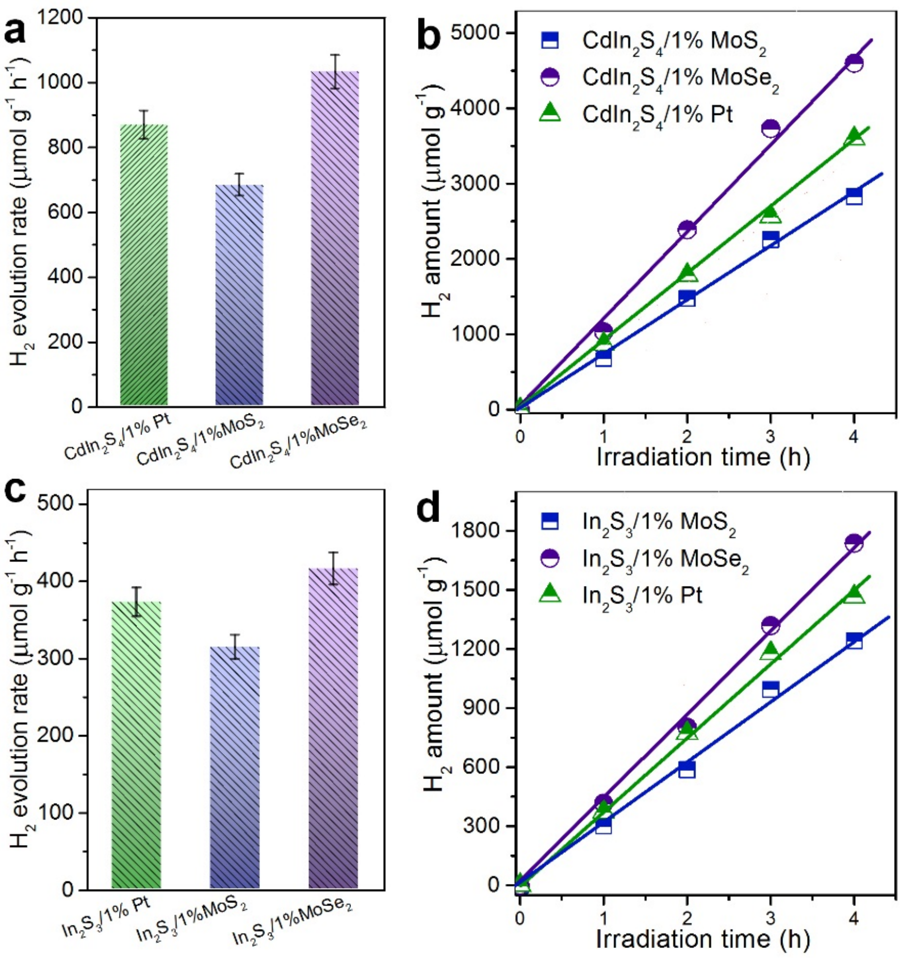

Supplementary Figure 44. Comparison of H_2 evolution activities of $CdIn_2S_4$/1%$MoSe_2$ and In_2S_3/1% $MoSe_2$ with reference photocatalysts, i.e., metal sulphide/Pt ($CdIn_2S_4$/1%Pt, In_2S_3/1%Pt,) and metal sulphide/MoS_2 ($CdIn_2S_4$/1% MoS_2, In_2S_3/1% MoS_2). Note: the error bars represent the photoactivity standard deviations calculated from triplicate experiments.

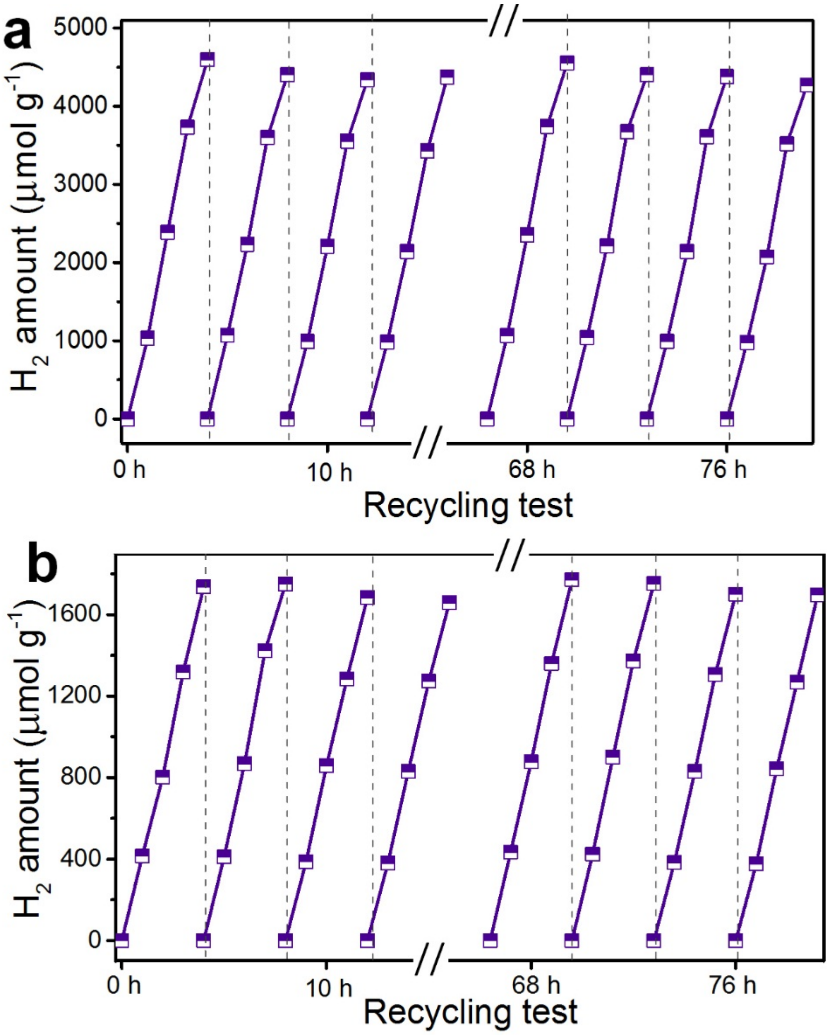

Supplementary Figure 45. Recycling of photoactivities over optimal (a) $CdIn_2S_4$/1%$MoSe_2$ and (b) In_2S_3/1%$MoSe_2$.

Supplementary Table 1. The comparison of photoelectrochemical performance of $ZnIn_2S_4/MoSe_2$ with some reported two-dimensional-based photocatalyst systems.

**Photocatalyst**	**Photocurrent density (μA cm^{-2})**	**Photocurrent Enhancement**	**Reference**
Bi_2WO_6 nanosheets	~ 0.5	ca. 10-fold of bulk Bi_2WO_6	*Nat. Commun.* **6**, 8340 (2015)[1]
Quantum Cu(II) nanodot/TiO_2 nanosheets	~ 7.8	ca. 9.8-fold of TiO_2 nanosheets	*J. Phys. Chem. C* **120**, 10746-10756 (2016)[2]
$SrNb_2O_6$ nanoplates	~ 3.3	ca. 8.3-fold of bulk Bi_2WO_6	*Chem. Commun.* **51**, 3430-3433 (2015)[3]
Au/TiO_2 Nanosheets/Au	~ 8.5	ca. 1.7-fold of TiO_2 nanosheets	*J. Phys. Chem. C* **116**, 6490-6494 (2012)[4]
Graphene-titania ($Ti_{0.91}O_2$) nanosheets	~ 0.5	ca. 5-fold of TiO_2 nanosheets	*Adv. Funct. Mater.* **19**, 3638-3643 (2009)[5]
CoO_x/TiO_2 nanosheets	~ 0.31	ca. 7.8-fold of TiO_2 nanosheets	*Appl. Catal. B* **190**, 44-65 (2016)[6]
Defect-rich ZnO nanosheets	~ 2.7	ca. 13-fold of ZnO nanoparticles	*Appl. Catal. B* **192**, 8-16 (2016)[7]
Oxygen-Doped $ZnIn_2S_4$ Nanosheets	~ 4.2	ca. 1.6-fold of pristine $ZnIn_2S_4$ nanosheets	*Angew. Chem. Int. Ed.* **55**, 6716-6720 (2016)[8]
SiC layer/graphene	~ 8	ca. 16-fold of SiC nanocrystals	*J. Mater. Chem. A* **3**, 10999-11005 (2015)[9]
BiOI nanosheets	~1.8	ca. 1.8-fold of BiOI plates	*Dalton Trans.* **43**, 9549-9556 (2014)[10]
Layered-titanate/RGO	~ 0.5	ca. 4-fold of titanate	*Small* **8**, 1038-1048 (2012)[11]
BiOCl sheet/carbon quantum dot	~ 0.22	ca. 3.3-fold of BiOCl sheet	*Nanotechnol.* **27**, 065701 (2016)[12]
Holey C_3N_4 nanosheets	~ 5.5	ca. 5.5-fold of bulk C_3N_4	*Adv. Funct. Mater.* **25**, 6885-6892 (2015)[13]
BiOBr nanosheets	~ 7	ca. 2.4-fold of BiOBr nanoplates	*Phys. Chem. Chem. Phys.* **16**, 20909-20914 (2014)[14]
C_3N_4 nanosheets	~ 2.1	ca. 7-fold of bulk C_3N_4	*J. Mater. Chem. A* **3**, 10999-11005 (2015)[15]
$ZnIn_2S_4/MoS_2$ hetero-layers	~ 5.0	ca. 22-fold of bulk $ZnIn_2S_4$	This work

Supplementary Note 1:

Dynamics analysis of emission decay for bulk $ZnIn_2S_4$ and $ZnIn_2S_4/MoSe_2$ hetero-layer structure.

$$I(t) = A_1 \bullet exp(-t/\tau_1) + A_2 \bullet exp(-t/\tau_2) \quad \mathbf{(1)}$$

$$\text{Ave.}\,\tau = \frac{A_1 \bullet \tau_1^2 + A_2 \bullet \tau_2^2}{A_1 \bullet \tau_1 + A_2 \bullet \tau_2} \quad \mathbf{(2)}$$

Supplementary References

1. Zhou, Y. *et al*. Monolayered Bi_2WO_6 nanosheets mimicking heterojunction interface with open surfaces for photocatalysis. *Nat. Commun.* **6,** 8340 (2015).
2. Zhang, M. *et al*. High H_2 evolution from quantum Cu(II) nanodot-doped two-dimensional ultrathin TiO_2 nanosheets with dominant exposed {001} facets for reforming glycerol with multiple electron transport pathways. *J. Phys. Chem. C* **120,** 10746-10756 (2016).
3. Xie, S., Wang, Y., Zhang, Q., Deng, W. & Wang, Y. $SrNb_2O_6$ nanoplates as efficient photocatalysts for the preferential reduction of CO_2 in the presence of H_2O. *Chem. Commun.* **51,** 3430-3433 (2015).
4. Wang, H., You, T., Shi, W., Li, J. & Guo, L. Au/TiO_2/Au as a plasmonic coupling photocatalyst. *J. Phys. Chem. C* **116,** 6490-6494 (2012).
5. Manga, K. K., Zhou, Y., Yan, Y. & Loh, K. P. Multilayer hybrid films consisting of alternating graphene and titania nanosheets with ultrafast electron transfer and photoconversion properties. *Adv. Funct. Mater.* **19,** 3638-3643 (2009).
6. Lu, D. *et al*. Visible light induced photocatalytic removal of Cr(VI) over TiO_2-based nanosheets loaded with surface-enriched CoO_x nanoparticles and its synergism with phenol oxidation. *Appl. Catal. B* **190,** 44-65 (2016).
7. Wang, J., Xia, Y., Dong, Y., Chen, R., Xiang, L. & Komarneni, S. Defect-rich ZnO nanosheets of high surface area as an efficient visible-light photocatalyst. *Appl. Catal. B* **192,** 8-16 (2016).
8. Yang, W. *et al*. Enhanced photoexcited carrier separation in oxygen-doped $ZnIn_2S_4$ nanosheets for hydrogen evolution. *Angew. Chem. Int. Ed.* **55,** 6716-6720 (2016).
9. Zhou, X. *et al*. Ultra-thin SiC layer covered graphene nanosheets as advanced photocatalysts for hydrogen evolution. *J. Mater. Chem. A* **3,** 10999-11005 (2015).
10. Mi, Y., Zhou, M., Wen, L., Zhao, H. & Lei, Y. A highly efficient visible-light driven photocatalyst: two dimensional square-like bismuth oxyiodine nanosheets. *Dalton Trans.* **43,** 9549-9556 (2014).
11. Kim, I. Y. *et al*. A strong electronic coupling between graphene nanosheets and layered titanate nanoplates: a soft-chemical route to highly porous nanocomposites with improved photocatalytic activity. *Small* **8,** 1038-1048 (2012).
12. Fang, D. *et al*. Fabrication of 2D sheet-like BiOCl/carbon quantum dot hybrids via a template-free coprecipitation method and their tunable visible-light photocatalytic activities derived from different size distributions of carbon quantum dots. Nanotechnol. **27,** 065701 (2016).

13. Liang, Q., Li, Z., Huang, Z.-H., Kang, F. & Yang, Q.-H. Holey graphitic carbon nitride nanosheets with carbon vacancies for highly improved photocatalytic hydrogen production. *Adv. Funct. Mater.* **25,** 6885-6892 (2015).
14. Chen, J., Guan, M., Cai, W., Guo, J., Xiao, C. & Zhang, G. The dominant {001} facet-dependent enhanced visible-light photoactivity of ultrathin BiOBr nanosheets. *Phys. Chem. Chem. Phys.* **16,** 20909-20914 (2014).
15. Qiu, P. *et al*. Fabrication of an exfoliated graphitic carbon nitride as a highly active visible light photocatalyst. *J. Mater. Chem. A* **3,** 24237-24244 (2015).
